# The Role of the Gut Microbiome in the Complex Network of Frailty Syndrome and Associated Comorbidities in Aging

**DOI:** 10.1111/acel.70365

**Published:** 2026-01-10

**Authors:** Ana Barberá, Rosario Ortolá, Mercedes Sotos‐Prieto, Fernando Rodríguez‐Artalejo, Andrés Moya, Susana Ruiz‐Ruiz

**Affiliations:** ^1^ Genomics and Health Department FISABIO València Spain; ^2^ CIBER of Epidemiology and Public Health (CIBERESP) Madrid Spain; ^3^ Department of Preventive Medicine and Public Health Universidad Autónoma de Madrid Madrid Spain; ^4^ IMDEA Food Institute CEI UAM+CSIC Madrid Spain; ^5^ Department of Environmental Health Harvard T.H. Chan School of Public Health Boston Massachusetts USA; ^6^ Institute for Integrative Systems Biology (I2SysBio) UV‐CSIC València Spain

**Keywords:** aging, Frailty syndrome (FS), microbiota, physical performance, resistome, sporulation

## Abstract

The gut microbiota changes throughout life, potentially influencing health and triggering physiological disorders. Frailty syndrome (FS) is an age‐related condition that reduces quality of life and increases hospitalization and mortality risks, making early detection and prevention essential in older populations. This study analyzed 16S rRNA gene and metagenomics sequencing of fecal samples from 203 older adults (FS: *n* = 64, non‐FS (NFS): *n* = 139) to assess the role of gut microbiota in FS and related comorbidities, such as sarcopenia and impaired lower extremity function (ILEF) or anthropometric variables. Consistent taxonomic patterns were observed: *Eggerthella*, *Parabacteroides*, and *Erysipelatoclostridium* were significantly abundant in FS, while *Christensenellaceae R‐7 group*, *Erysipelotrichaceae UCG‐003*, and *Hungatella* were enriched in NFS. *Christensenellaceae R‐7 group* was also associated with better mobility. Metagenomics analysis identified 680 KEGG functions differing between groups, categorized into 28 metabolic pathways. FS individuals had overrepresented biotin metabolism, antimicrobial resistance, and energy production, but underrepresented ribosomal and protein synthesis and sporulation pathways. Resistome analysis found the tetM/tetO (K18220) gene most abundant, alongside tetracycline, β‐lactam, and macrolide resistance, primarily mediated by antibiotic efflux and transporters. These findings highlight distinct microbial and functional signatures associated with FS, underscoring the complex interplay between the gut microbiota and host physiology in aging. Adjusting for covariates, age and diabetes acted as confounding factors in FS for both 16S gene and metagenomics sequencing. This study offers new insights into fundamental questions in the biology of aging and opens avenues for microbiota‐targeted strategies to improve the quality of life in older adults.

## Graphical abstract

This study analyzed gut microbiota in older adults with and without Frailty Syndrome by 16S rRNA gene sequencing and metagenomics. Frailty was associated with altered microbial composition, metabolic functions, and increased antimicrobial resistance, whereas non‐frail individuals showed healthier profiles. Some studied comorbidities acted as confounding factors in both taxonomy and function influencing microbiota–frailty associations.

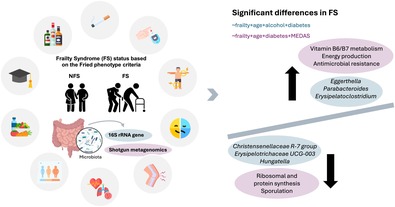

## 1 Introduction

Frailty syndrome (FS) is a multidimensional clinical condition characterized by increased vulnerability to adverse health outcomes due to the cumulative decline in physiological reserve and function in older adults. Considering that FS is a heterogeneous syndrome, the standardization of a diagnostic method is challenging. It can be identified either through a physical phenotype (Fried et al. 2001)—marked by weakness, weight loss, exhaustion, slowness, and low activity—or by the accumulation of health deficits across multiple systems, reflecting biological aging and diminished resilience to stressors (Mitnitski et al. 2001). Importantly FS is a state that precedes disability, and its early detection and prevention are crucial to mitigate its detrimental clinical consequences. In fact, frail individuals are more prone to experience a series of adverse clinical events after exposure to even small stressors. Several physiological mechanisms are more pronounced in patients with FS compared to healthy elderly individuals, including chronic inflammation, redox balance alteration, mitochondrial dysfunction, genomic damage, loss of proteostasis, and metabolic alterations (Davinelli et al. 2021). These changes contribute to dysfunction in nervous, neuroendocrine, cardiovascular, respiratory, renal, immune, and musculoskeletal systems (Khan et al. 2019; Rodríguez‐Mañas et al. 2013).

The rapid growth of the elderly population in the world has contributed to an increasing prevalence of FS, presenting substantial challenges for the healthcare systems and the whole society (Dent et al. 2019). Chronic conditions, malnutrition, reduced physical activity, and social isolation can contribute to its development, together with the high incidence of comorbidities such as cardiovascular disease, diabetes, and cognitive impairment, further increasing susceptibility to FS (Rodríguez‐Mañas et al. 2013). The prevalence of FS among the global older population is estimated to be between 4% and 27%, increasing with age (Khan et al. 2019). Despite the widespread nature of FS, it is frequently underdiagnosed. The healthcare system predominantly focuses on managing acute illnesses, whereas FS requires a comprehensive, multidisciplinary approach that addresses not only physical but also psychological and social factors (O'Caoimh et al. 2018).

The gut microbiome, also referred to as “the second genome,” undergoes dynamic changes throughout aging. Certain bacterial taxa and their associated metabolic functions could influence health status and trigger physiological disorders (Guo et al. 2022). The microbiota carries out functions that human cells cannot perform and plays a crucial role in human metabolism, the development of the immune system, the prevention of infections, and even the proper functioning of the brain and nervous system. These microbial communities are dynamic and can change in response to various factors, including diet, disease, and antibiotic use, but also change across the lifespan. In older adults, the diversity of gut microbiota tends to decrease, which can lead to an imbalance in microbial communities (Ragonnaud and Biragyn 2021; Schoultz et al. 2025), despite some studies reporting conflicting trends regarding age‐related diversity (Badal et al. 2020). This imbalance contributes to a chronic, low‐grade inflammatory state termed “inflammaging,” which is thought to be key to FS (Claesson et al. 2012). Several studies have demonstrated that frail individuals tend to have less microbial diversity and a lower abundance of beneficial bacteria, such as *Bifidobacterium* and *Lactobacillus* species (O'Toole and Jeffery 2015). Moreover, the gut microbiota is closely linked to nutritional status, and poor dietary habits often associated with FS can further disrupt the microbial balance.

Emerging evidence suggests the relationship between gut microbiota and FS development. van Tongeren et al. (2005) reported significant alterations in the microbiota composition of older adults with FS, characterized by reduced levels of *Bacteroides*/*Prevotella*, *Lactobacillus*, and *Faecalibacterium prausnitzii* and increased levels of the Enterobacteriaceae family. Claesson et al. (2012) demonstrated that healthier older adults tend to have a microbial profile similar to younger adults and exhibit better results in pro‐inflammatory markers and cognitive function tests. Further studies by Ghosh et al. (2020) and Ye et al. (2023) suggest that fiber‐rich dietary interventions, particularly those based on the Mediterranean diet, can modulate the gut microbiota in both mice and humans with FS, shifting the microbial profile toward that of non‐frail individuals and reducing inflammatory markers associated with FS.

These findings underscore the complex interplay between the gut microbiota, diet, and aging, suggesting potential management avenues for FS by modulating the microbiota. Probiotic and prebiotic supplements, along with dietary changes promoting gut health, are being investigated as strategies to mitigate FS (Sánchez et al. 2022). While the relationship between FS and gut microbiota is still being explored, understanding the mechanisms behind this interaction holds promise for developing therapeutic strategies that could improve the health and quality of life for older adults worldwide. Accordingly, this article aims to identify and analyze gut microbiota taxa related to FS and the functional analysis of their genes among older adults from Spain.

## 2 Materials and Methods

### 2.1 Study Design and Participants

Fecal samples were obtained from participants in the third wave of the Seniors‐ENRICA‐2 cohort, which includes community‐dwelling individuals aged between 70 and 86 years, holding a national healthcare card, and living in two districts of the city of Madrid (Spain) and four surrounding large towns (Ortolá et al. 2022; García‐Esquinas et al. 2021). Socio‐demographic, lifestyle, and morbidity data were collected through a computer‐assisted telephone interview, followed by two home visits for a physical examination, dietary history acquisition, and biological samples collection. The information was obtained with questionnaires, scales, and performance tests validated or widely used in the literature. All individuals provided written informed consent and collected a fecal sample in sterile tubes containing 10 mL of RNAlater Solution (Invitrogen).

In this study, approved by the Ethics Committee of FISABIO (Cod: 20230127/08), a sub‐sample of 203 individuals (ENRICA‐EE) was selected according to their frailty status (FS and non‐frailty status, NFS). FS was defined, based on the Fried phenotype, as having three or more of the following five criteria (Fried et al. 2001): (i) exhaustion, evaluated as responding “≥ 3 days a week” to either of the following two questions from the Center for Epidemiologic Studies Depression Scale (Ruiz‐Grosso et al. 2012): “I felt that anything I did was a big effort” or “I felt that I couldn't keep on doing things”; (ii) muscle weakness, defined as maximum grip strength on the dominant hand ≤ 30 kg in men and ≤ 18 kg in women, measured with a Jamar dynamometer (Ottenbacher et al. 2002; García‐García et al. 2011); (iii) low physical activity, defined as walking ≤ 2.5 h per week in men and ≤ 2 h in women; (iv) slow walking speed, defined as taking ≥ 3 s to complete the 3‐m walking speed test (Guralnik et al. 1994); (v) unintentional weight loss, ≥ 4.5 kg of body weight lost in the preceding year. To align with our study population and available data, we adopted slight modifications of the Fried definition: using absolute rather than population‐based cutoffs for muscle weakness and slow walking speed to allow for direct comparison across different cohorts and settings, especially when population‐specific distributions differ widely, and using walking time rather than calculating total energy expenditure across multiple physical activities, because it is easier to recall and report accurately, especially in older populations, and reflects overall mobility and endurance.

Among other comorbidities, sarcopenia and impaired lower extremity function (ILEF) were also assessed due to their common association with FS and aging. The Short Physical Performance Battery score (SPPB) (Guralnik et al. 1994) was used to evaluate the lower extremity function, combining the results of three tests: gait speed, chair stand, and balance. Scores from 0 to 9 were considered as ILEF, while 10–12 indicated a better performance. Sarcopenia was defined as the presence of low muscle mass (appendicular skeletal muscle mass measured with bioelectrical impedance analysis < 20 kg in men and < 15 kg in women) plus low muscle strength (average hand grip strength < 27 kg in men and < 16 kg in women) and/or low physical performance (> 15 s for five rises in the chair stand test) (Cruz‐Jentoft et al. 2019). Lifestyle variables were also considered; participants were classified as “never”, “former” or “current” smokers, and alcohol consumption was categorized as “weekly,” “monthly,” or “rare.”

### 2.2 Sample Preparation

Upon arrival, all fecal samples were processed on the same day in a Class II biological safety cabinet under sterile conditions. The samples were then homogenized by vigorous mixing with 10 mL of phosphate‐buffered saline (PBS), followed by centrifugation for 5 min at 2000 rpm and 4°C to remove any solid debris. The resulting microbial suspension was stored at −80°C for future analysis.

### 2.3 DNA Isolation and Purification

Genomic DNA was isolated from 500 μL of fecal microbial suspension using the QIAamp Fast DNA Stool Mini Kit (QIAGEN), incorporating both enzymatic and mechanical lysis steps to ensure efficient disruption of bacterial cell walls. Samples were pre‐treated with lysozyme (20 μL, 37°C, 30 min), followed by bead‐beating with 100 μL glass beads and heat at 95°C during 5 min. Samples were then centrifuged, and the supernatant was transferred to new tubes containing 45 μL of proteinase K. The protocol continued following the manufacturer's instructions. DNA concentrations were determined using the Qubit DNA HS Assay Kit (Thermo Fisher Scientific).

### 2.4 16S rRNA Gene Amplification, Library Preparation and Sequencing

The V3–V4 hypervariable regions of the 16S rRNA gene were amplified from extracted DNA following the 16S Metagenomic Sequencing Library Preparation Illumina protocol. Extraction controls were amplified and sequenced in parallel with the samples. PCR amplification was done with primers: 5′‐TCGTCGGCAGCGTCAGATGTGTATAAGAGACAGCCTACGGGNGGCWGCG‐3′ (forward) and 5′‐GTCTCGTGGGCTCGGAGATGTGTATAAGAGACAGGACTACHVGGGTATCTATCC‐3′ (reverse). Amplicons were purified using AMPure XP beads (Macherey‐Nagel). Then, Illumina sequencing adaptors and dual‐index barcodes (Nextera XT v2 index kit) were added to the amplicons using an 8‐cycle PCR. Libraries were pooled at equimolar concentrations and sequencing was done using a paired‐end, 2 × 300 pb cycle run on an Illumina NextSeq2000 following the manufacturers' recommendations.

### 2.5 Metagenomic Library and Sequencing

Whole‐genome sequencing was conducted using the total DNA extracted from the fecal samples. Metagenome libraries were obtained with Illumina's Nextera XT DNA Library Preparation Kit: input DNA (0.2 ng/μL) was tagmented (tagged and fragmented simultaneously) by the kit transposome and the unique barcodes, allowing amplification by PCR in subsequent steps. (Macherey‐Nagel), PCR products were purified using AMPure XP beads (Macherey‐Nagel) and excess contaminants were removed by washing with 80% ethanol. An equimolar library pool was prepared and sequenced using Illumina NextSeq 2 × 300 pb paired‐end reagent P2 kit on a NextSeq2000 system, according to the manufacturer's instructions.

### 2.6 Data Processing

For 16S and metagenomics analysis, raw reads were quality checked by the use of the Fastp program (Chen et al. 2018) and mapped against the human genome using Bowtie2 (Langmead and Salzberg 2012) to remove nonspecific amplification reads or those of human origin usually appearing in metagenomic samples. All samples passed quality control thresholds, including minimum sequencing depth (at least 10^5^ reads for 16S and 10^6^ for metagenomics) and metadata completeness. Therefore, no samples were excluded from the analyses.

#### 2.6.1 16S rRNA Amplicons

16S taxonomic profiling was performed using the DADA2 package (v1.20.0) in R (Callahan et al. 2016). PCR primers were removed with the cutadapt routine, and sequences were quality‐checked using DADA2 v.1.20 (Callahan et al. 2016). Reads were trimmed to remove bases with quality scores below 20 and those shorter than 100 bases, with entropy lower than 70. The V3‐V4 primers were trimmed and reads with more than five expected errors were discarded. All samples passed quality control thresholds. Amplicon Sequence Variants (ASVs) were built by estimating base transition error rates, dereplicating reads, and merging forward and reverse pairs with at least 15 overlapping bases. Taxonomy was assigned to each ASV by aligning against the Silva v.138.1 database using Blastn with ≥ 95% identity and 100% coverage. The best alignment (≥ 97% identity) was selected, and the species of this alignment was assigned to the corresponding ASV. All samples achieved the plateau in rarefaction curves.

#### 2.6.2 Metagenomic Analysis

To assess the taxonomy, by means of Kaiju (v.1.9.2) (Menzel et al. 2016), using the NCBI nr + euk reference database (10th May 2023), with a maximum of 5 mismatches allowed and a minimum matching length of 25 amino acids. For the functional assignment, the shotgun metagenomic sequences passing the quality assessment and non‐human reads were analyzed with the SqueezeMeta v.1.6.2 pipeline (Tamames and Puente‐Sánchez 2019). The software FLASH was used to join overlapping pairs and obtain longer sequences, and sequential metagenome assemblies were done with Megahit to obtain contigs of DNA sequences from each sample and annotated against the Kyoto Encyclopedia of Genes and Genomes (KEGG) database.

#### 2.6.3 Resistome Analysis

To elucidate the role of gut resistome in FS, the amino acid SqueezeMeta ORF sequences were aligned against the canonical CARD database (v.3.2.8; 23rd October 2023), by means of the RGI tool (v.6.0.3; 21st September 2023) (Alcock et al. 2023), with input type parameter equal to “protein”. Simultaneously, ORF sequences were also analyzed by means of the AMRFinderPlus tool (v.3.11.26; 15th November 2023) against the Reference Gene Catalog database (Feldgarden et al. 2021), with “protein” and “plus” parameters. Only the members of each read pair showing at least 80% sequence identity and 70% read coverage against the hit sequence were selected.

### 2.7 Statistical Analysis

Descriptive statistics were run on all data. Differences between FS and NFS participants were assessed via the Wilcoxon or Fisher exact test for continuous and categorical variables, respectively.

The effect of frailty on taxonomy and functional ortholog gene abundances was first modeled using the formula ~*frailty*. From 16S sequences information, alpha diversity was estimated with the Shannon diversity index and Chao1 richness estimator (vegan library, R), while beta diversity was evaluated with the Bray–Curtis dissimilarity and visualized by Principal Coordinates Analysis (PCoA). Normalization data and differentially abundant bacterial taxa were identified with the ANCOM‐BC package (Lin and Peddada 2020), and distinctive microbial patterns were assessed using PERMANOVA. The Boruta random forest algorithm (Kursa and Rudnicki 2010) was applied to confirm differentially abundant taxa. Functional differential analyses of metagenomic reads (transcripts per million) were performed using the DESeq2 pipeline, followed by enrichment analysis grouping significant KEGG functions into metabolic pathways through a hypergeometric distribution. Correlation analyses for both normalized 16S and metagenomic read data and continuous clinical variables were performed using the mixOmics sPLS‐canonical approach (Lê Cao et al. 2011). For associations involving categorical clinical variables, PERMANOVA tests were conducted using Bray‐Curtis dissimilarities and results were visualized using PCoA plots.

A sensitivity analysis was performed in alpha diversity using linear mixed‐effects models (nlme package, R) with the formula *~frailty* + *variable* to evaluate the potential confounding effect of anthropometric and clinical variables that differed between FS and NFS groups (Table 2). For beta diversity, sensitivity analyses were conducted using PERMANOVA (Bray–Curtis dissimilarity) with the model ~ *frailty* + *variable* + *frailty* × *variable* to assess both confounding and possible effect modification. A multivariate PERMANOVA analysis was done including all FS criteria simultaneously (*~BCdissimilarity* + *exhaustion* + *weight loss* + *low activity* + *reduced gait speed* + *grip strength*), to evaluate the independent association of each criterion with taxonomic and functional microbiome profiles. All results are reported in Table S1.

All analyses were performed in R. Statistical significance was defined as adjusted *p*‐values < 0.05 after Benjamini–Hochberg correction, except for enriched metabolic pathways, where raw *p*‐values were provided.

## 3 Results

### 3.1 Description of the Study Participants

Fecal samples were obtained from 203 participants in the Seniors‐ENRICA‐2 cohort, 103 females (76.56 ± 4.74 years) and 100 males (75.21 ± 4.25 years). Based on FS criteria, the individuals were categorized into 64 FS and 139 NFS (Table 1). Clinical data from 201 individuals (Table 2) revealed a strong association between FS and comorbidities. Osteoarthritis was the most prevalent co‐occurrence condition in FS individuals, followed by diabetes, cardiovascular disease, and depression (*p*‐values: 7.33e‐07, 0.0015, 0.0036, and 0.0015, respectively). Respiratory conditions were slightly more frequent in NFS, but differences were not significant (*p* = 0.78), suggesting that not all chronic conditions contribute equally to FS. Tobacco and alcohol use were more frequently reported in NFS, although only alcohol consumption differed significantly between groups (*p* = 6e‐04). Sarcopenia, a key aging‐related condition, was notably more prevalent in FS individuals (14 FS vs. 1 NFS), similar to the individuals with impaired lower extremity function (ILEF) (47 FS vs. 5 NFS).

**TABLE 1:** Descriptive data of study participants. The number of cases (no.) and percentage of cases (%) are indicated within each age group.

	Total	FS			NFS		
		Total	Male	Female	Total	Male	Female
*N*	203	64	15	49	139	85	54
Mean age ± σ (years)	75.9 ± 4.5	78.7 ± 4.5	79.1 ± 4.4	78.6 ± 4.6	74.6 ± 3.9	74.5 ± 3.8	74.7 ± 4.1

Age (years)	Total no. (%)	Total no. (%)	Male no. (%)	Female no. (%)	Total no. (%)	Male no. (%)	Female no. (%)
70–74	89 (43.8)	13 (20.3)	3 (20)	10 (20.4)	76 (54.7)	47 (55.3)	29 (53.7)
75–78	58 (28.6)	17 (26.6)	4 (26.7)	13 (26.5)	40 (28.8)	26 (30.6)	15 (27.8)
79–82	36 (17.7)	19 (29.7)	6 (40)	13 (26.5)	17 (12.2)	10 (11.8)	7 (12.9)
≥ 83	20 (9.8)	15 (23.4)	2 (13.3)	13 (26.5)	5 (3.6)	2 (2.3)	3 (5.6)

**TABLE 2:** Clinical information from 201 individuals out of the 203 included in the study.

Features	FS (*n* = 64)	NFS (*n* = 137)	*p*‐value
Age—mean ± *σ*	78.7 ± 4.5	*n* = 139 74.6 ± 3.9	0.00019
Sex—no. (%)		*n* = 139	
Male	15 (23.44%)	85 (61.15%)	5.92e‐06
Female	49 (76.56%)	54 (38.85%)	
Tobacco—no. (%)			
Never	44 (68.8%)	68 (49.6%)	0.084
Former	15 (23.4%)	56 (40.9%)	
Current	5 (7.8%)	13 (9.5%)	
Alcohol—no. (%)			
Weekly	18 (28.1%)	80 (58.4%)	6.00e‐04
Monthly	5 (7.8%)	18 (13.1%)	
Rare	39 (60.9%)	37 (27.0%)	
Education level—no. (%)			
Primary	43 (67.2%)	73 (53.3%)	0.096
Secondary	14 (21.9%)	24 (17.5%)	
University	7 (10.9%)	40 (29.2%)	
BMI (kg/m^2^)—mean ± *σ*	28.76 ± 4.9	26.4 ± 3.13	0.003
Energy (kcal/day)‐ mean ± *σ*	1790.2 ± 259.2	1934.8 ± 247.1	0.0053
MEDAS—mean ± *σ*	7.31 ± 1.4	8.04 ± 1.66	0.055
Diabetes—no. (%)	19 (29.7%)	13 (9.5%)	0.0015
Respiratory disease—no. (%)	8 (12.5%)	13 (9.5%)	0.78
Cardiac disease—no. (%)	16 (25%)	11 (8.03%)	0.0036
Depression—no. (%)	12 (18.8%)	5 (3.8%)	0.0015
Cancer—no. (%)	10 (15.6%)	10 (7.6%)	0.11
Osteoarthritis—no. (%)	42 (65.6%)	37 (27.0%)	7.33e‐07
Rheumatoid arthritis—no. (%)	7 (10.9%)	10 (7.3%)	0.38
Sarcopenia—no. (%)	14 (21.8%)	1 (0.73%)	1.2e‐06
ILEF—no. (%)	47 (73.4%)	5 (3.6%)	4.05e‐25

*Note:* Adjusted *p*‐value resulted from the Wilcoxon or Fisher test. The number of cases (no.) and percentage of cases (%) are indicated within each group for categorical clinical data. Mean ± standard deviation (*σ*) is shown for continuous clinical data.

Abbreviations: BMI, Body Mass Index; ILEF, impaired lower‐extremity function; considered when the SPPB score is < 9; MEDAS, Mediterranean Diet Adherence Screener; considering the higher score (from 0 to 14), the greater the adherence.

### 3.2 Impact of FS on Fecal Microbiota Composition

**FIGURE 1:**
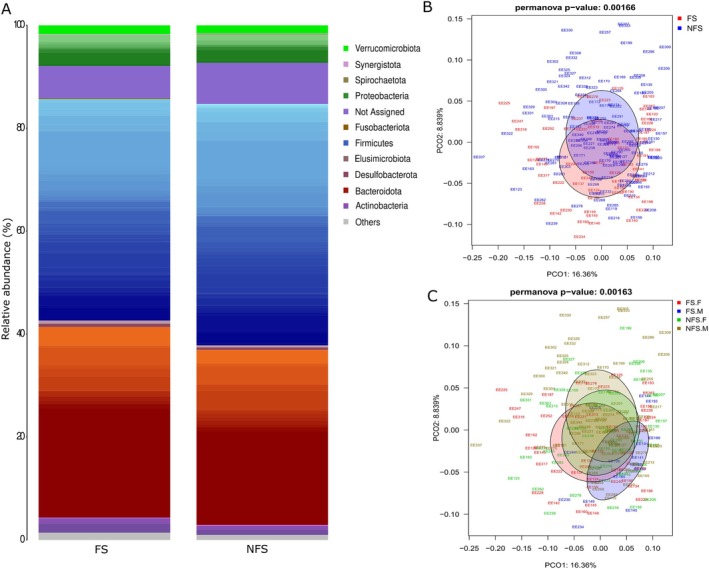
(A) Taxonomic composition at the genus level was found for each group (FS, NFS). The *y*‐axis represents the relative abundance assigned to each genus colored by phyla. (B) Principal Coordinates Analysis (PCoA) based on Bray‐Curtis distances of FS and NFS groups comparison and (C) gender‐grouped samples at the genus level. FS, frail, NFS, non frail.

For the 16S rRNA gene, alpha diversity analysis (Chao1) revealed higher genus‐level richness in the FS group compared to NFS (*p* = 0.0015; Figure S1A), notably between FS females and males (*p* = 0.044), and between frail and non‐frail males (*p* = 2.55e‐5; Figure S1B). No significant differences were found using the Shannon index (Figure S1C). Thus, the microbiota in the FS group presented higher richness but was uneven, especially for the frail male group. Beta diversity analyses at genus level showed significant differences in the gut microbiota structure between the FS and NFS groups, with significant sex‐specific differences (PERMANOVA *p* < 0.01; Figure 1B,C). Notably, group comparisons showed that all combinations involving FS were significant, except between NFS males and females.

To account for potential confounding factors, we next adjusted for covariates. The effect of age, alcohol, and diabetes was statistically significant (*p* = 0.0133, 0.041, 0.0133) (Table S1B), indicating that the effect of FS on gut microbiota composition was also explained by these covariates. The results for the FS criteria showed that exhaustion (*p* = 0.033) and weight loss (*p* = 0.041) were each independently associated with the bacterial composition, whereas grip strength (*p* = 0.386), gait speed (*p* = 0.474), and low activity (*p* = 0.064) did not show independent associations and may overlap with other criteria (Table S1C).

Due to the comorbidity of study participants, additional differential abundance analyses were conducted to assess whether other pathologies mentioned in Table 2 might affect the gut microbiota composition in our cohort without considering FS status. Significant microbiota shifts were only associated with diabetes (*p* = 0.0022, Figure S2). No differences were detected for other clinical variables.

**FIGURE 2:**
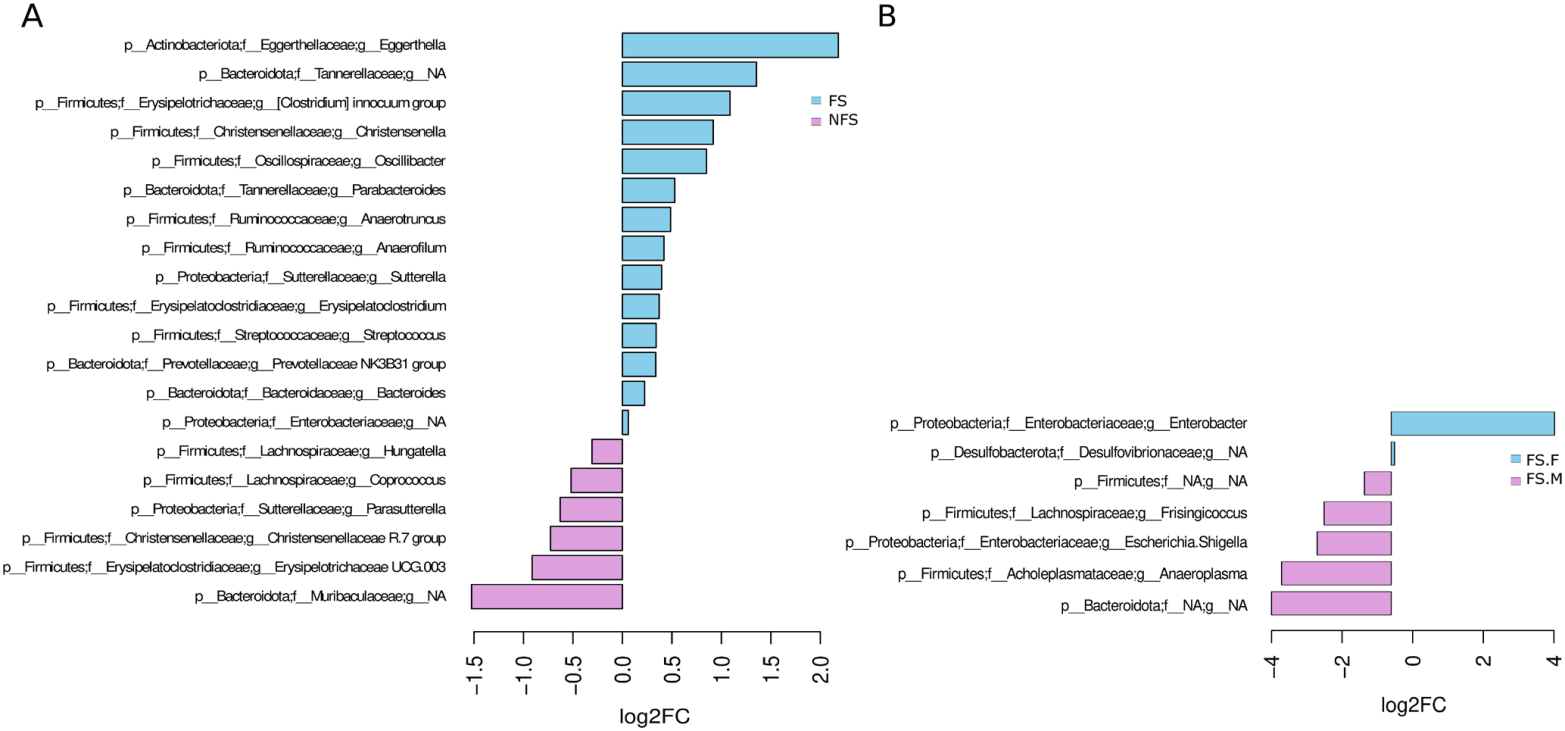
Barplot of differentially abundant taxa in (A) FS compared to NFS group and (B) frailty individuals (FS.F vs. FS.M) performed by ANCOM‐BC test and confirmed by Boruta algorithm. Taxonomic levels were indicated as p, phylum; f, family; g, genus. FS, frail, NFS, non‐frail.

The phylum profiles in both groups FS and NFS were similar (Figure 1A). However, *Firmicutes* was significantly more abundant in the NFS group (*p* = 0.018), while *Fusobacteriota* was significantly higher in the FS group (*p* = 0.014) (Table S2). Differential abundance analysis between the FS and NFS groups conducted using the ANCOM‐BC test revealed 23 bacterial genera with statistical differences in the inter‐group comparison, with 20 confirmed by Boruta feature selection (Figure 2A, Table S2B). *Eggerthella* became significantly more abundant in the FS group than in the NFS group. Other genera within Firmicutes, such as *Clostridium innocuum* , *Christensenella*, *Oscillibacter*, *Anaerotruncus*, *Anaerofilum*, *Erysipelatoclostridium*, and *Streptococcus*, were higher in FS. Additionally, *Bacteroides*, *Parabacteroides*, *Sutterella*, and the *Prevotellaceae NK3B31 group* also showed increased abundance in FS. Interestingly, the family Muribaculaceae‐associated group and the genera *Erysipelotrichaceae UCG‐003*, *Christensenellaceae R7 group*, *Parasutterella*, *Coprococcus*, and *Hungatella* were more abundant in the NFS group. When we compared FS.F and FS.M groups (Figure 2B), only seven taxa were confirmed by Boruta. However, five taxa presented high differences between groups (Log2FC > ±2). The genus *Enterobacter* and family Desulfovibrionaceae were increased in frail females, while the genera *Frisingicoccus*, *Anaeroplasma*, *Escherichia‐Shigella*, and not assigned genera from Firmicutes and Bacteroidota phyla showed higher abundance in FS males. The previously observed differences in the overall microbiota composition among sex‐grouped individuals with FS might be influenced by these specific taxa.

**FIGURE 3:**
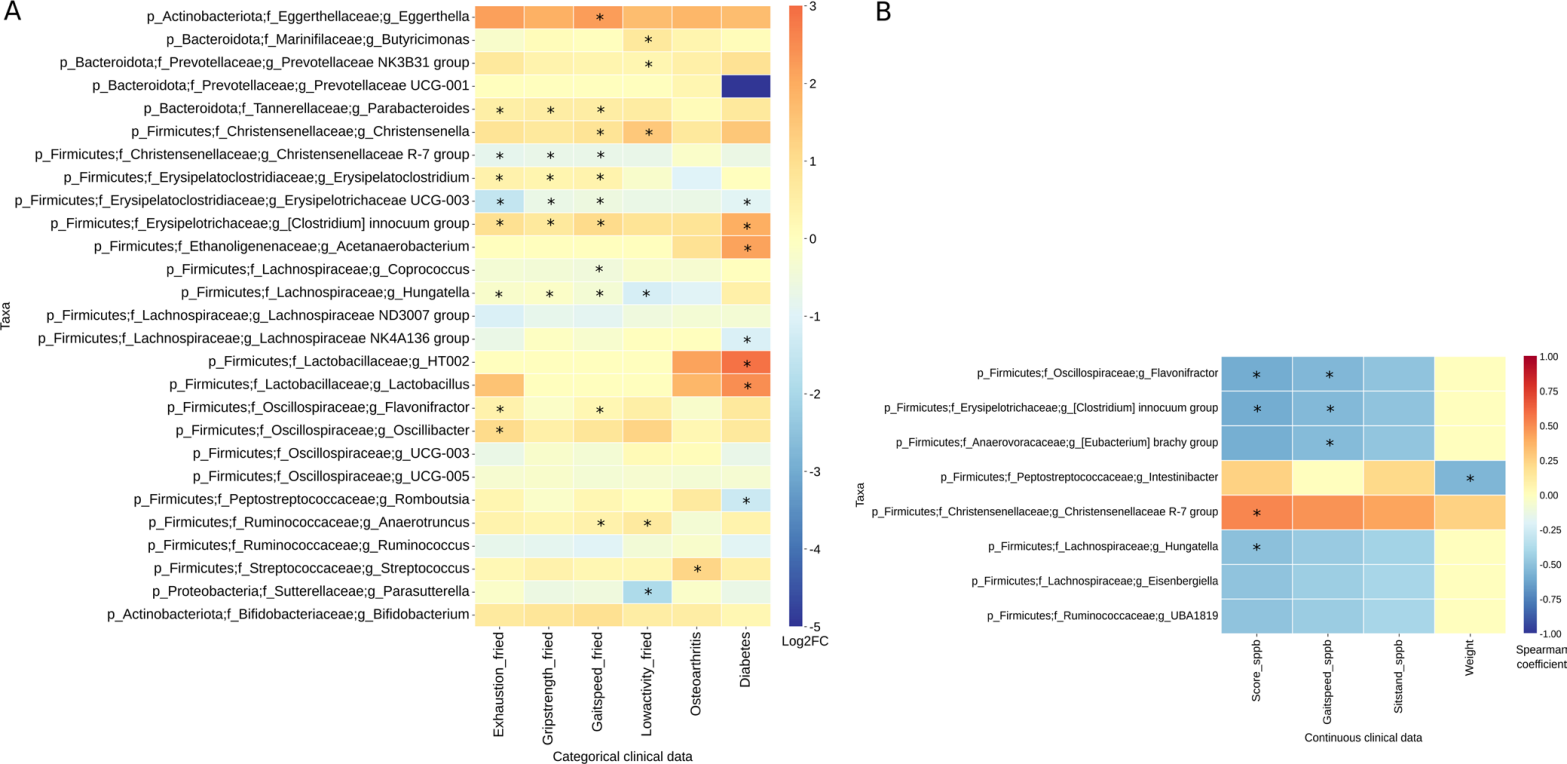
(A) Heatmap of Log2FC values of differentially abundant taxa for each categorical clinical feature. Significant differences performed by the ANCOM‐BC test (*p* < 0.05) and confirmed by Boruta were marked with an asterisk (*). Positive values indicate an increase in the taxon among individuals meeting the feature, while negative values indicate a decrease in the taxon compared with the individuals not meeting the feature. (B) Heatmap of the correlation between taxa abundances and continuous clinical features with a Spearman correlation coefficient equal to or greater than ±0.5. Statistically significant correlations were marked with an asterisk (*). Taxonomic levels were indicated as p, phylum; f, family; g, genus.

Taxonomic differences were observed for the four FS criteria, as well as for osteoarthritis and diabetes; however, no specific taxa were differentially abundant in sarcopenia. Notably, five genera were enriched in individuals with exhaustion, while three were consistently depleted. Similar patterns were observed for grip strength, reduced gait speed and low physical activity. This analysis suggests that while some genera are uniquely associated with specific clinical features, others like *Parabacteroides*, *Erysipelatoclostridium*, *Christensenellaceae R‐7 group*, *Erysipelotrichaceae UCG‐003*, and *Hungatella* showed consistent patterns across multiple frailty‐related features (Figure 3A).

We assessed correlations between 16S taxonomy and continuous clinical data related to lower limb function (ILEF) and weight (Figure 3B, Table S4). Lower physical performance—reflected by reduced SPPB scores, gait speed, and sit‐to‐stand test—was negatively correlated with six genera, indicating that higher abundances of these taxa are associated with poorer physical performance at the lower limbs (*ρ* < −0.5 for SPPB score). Only *Flavonifractor*, *Clostridium innocuum group*, *Eubacterium brachy* group, and *Hungatella* showed statistical significance (Figure 3B). In contrast, the *Christensenellaceae R‐7* group was positively correlated with the SSPB score (ρ = 0.53; *p* = 0.0355). *Intestinibacter* was negatively correlated with weight (*ρ* = −0.55; *p* = 0.0003), but showed weak positive associations with both SPPB score (*ρ* = 0.25; *p* = 0.054) and sit‐to‐stand test (*ρ* = 0.22; *p* = 0.16), implying a possible link of both *Christensenellaceae R‐7* group and *Intestinibacter* to better mobility. As lower SPPB scores indicate impaired lower limb function (ILEF), these results support associations between specific gut microbes and physical performance in aging.

### 3.3 Shotgun Metagenomics

#### 3.3.1 Taxonomic Study Beyond Bacteria

A total of 5.6e+08 paired‐end reads and an average of 2.7 (±0.83) million reads per sample were obtained. Non‐bacterial taxa were minimally represented, with only 0.049% of reads assigned to Eukarya and 4.011% to viral sequences. The viral taxa with the higher abundances were the *Caudoviricetes* class and *Microviridae* and *Inoviridae* families, all three corresponding to phage viruses that infect prokaryotes. Differential abundance analysis by ANCOM‐BC revealed *Inoviridae* as the only family significantly differing between FS and NFS (*p* = 0.007; log2fc = −0.45), with a higher abundance in the NFS group.

#### 3.3.2 Functional Analysis

The PERMANOVA test of functional data showed significant differences between FS and NFS groups (*p* = 0.00166) and among FS individuals grouped by sex (*p* = 0.0366), but not within the NFS group (*p* = 0.215) (Figure S4). When adjusting for covariates, age, MEDAS, and diabetes had statistically significant interactions with FS (*p* = 0.019, 0.023, 0.004) (Table S1D). The FS criteria exhaustion (*p* = 0.216) and weight loss (*p* = 0.471), grip strength (*p* = 0.370), gait speed (*p* = 0.658), and low activity (*p* = 0.509) did not show independent associations and may overlap with other criteria (Table S1E).

Significant differences in bacterial functional profiles were also linked to comorbidities such as diabetes (*p* = 0.001) and alcohol consumption (*p* = 0.011) (Figure S3); but no significant differences were observed for other categorical clinical features. These results corroborate the previous taxonomic finding and suggest that FS is associated with both compositional and functional shifts in the gut microbiome.

**FIGURE 4:**
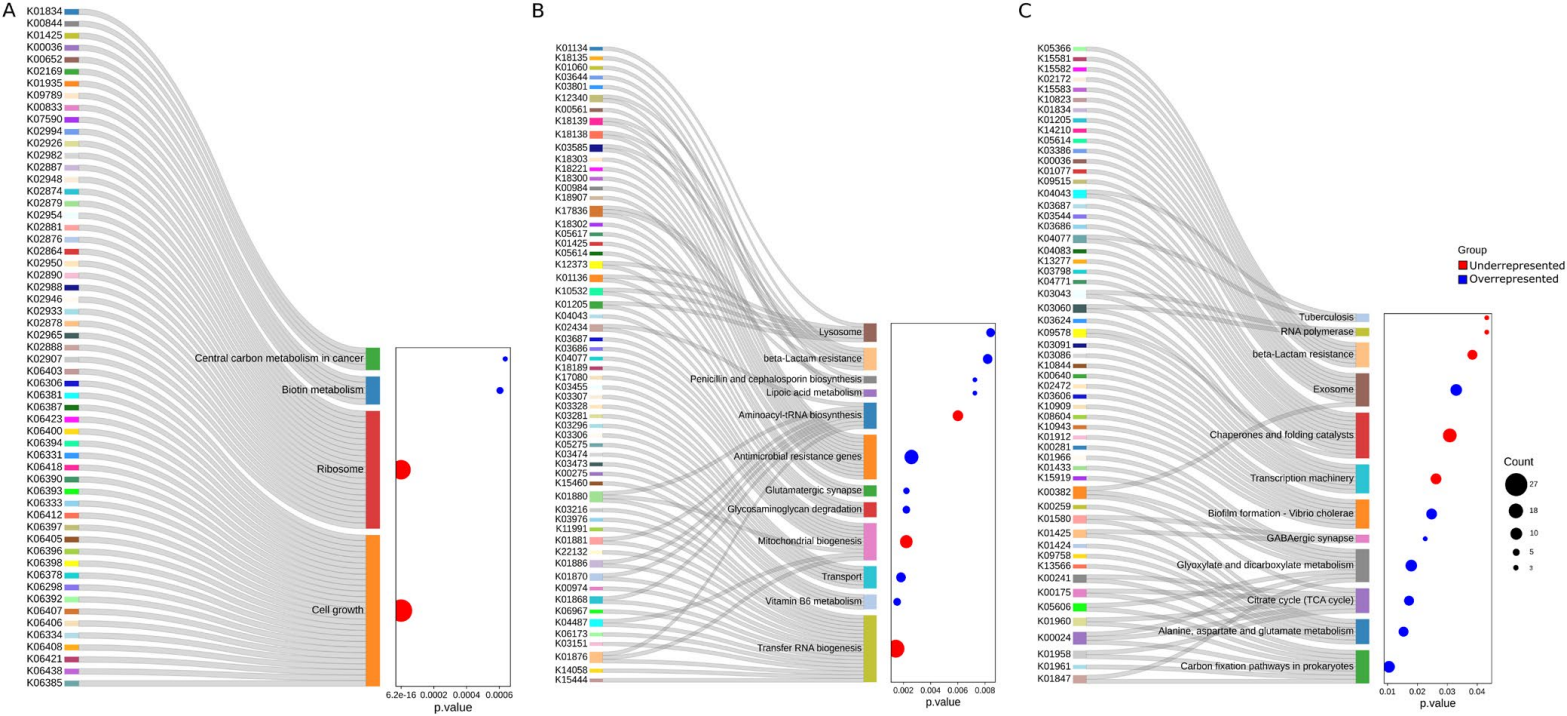
Sankey‐dotplot of the statistically different pathways identified through an enrichment analysis conducted using KEGG functions with adjusted *p*‐value < 0.05 in a DESeq2 test between FS and NFS groups. Pathways were divided into different plots depending on their statistical significance: *p* < 0.001 (A), *p* < 0.01 (B) and *p* < 0.05 (C). Overrepresented functions in the FS group are colored blue, while underrepresented functions in the FS group are colored red. FS, frail; NFS, non frail.

Differential analysis using DESeq2 identified 680 KEGG functions with significant differences between FS and NFS groups (*p* < 0.05). Of these, the enrichment analysis identified 209 KEGG functions grouped into 28 metabolic pathways statistically different (Figure 4). Among the overrepresented pathways in the FS group, very high significance (*p* < 0.001) was found in biotin (vit. B7) metabolism (*p* = 0.0006) and central carbon metabolism in cancer (*p* = 0.0006). Several KEGG were involved in the BioU and BioI biotin biosynthesis pathways (Figure 4A), leading to the production of desthiobiotin, a key precursor of biotin involved in the breakdown of fats, carbohydrates and proteins. Conversely, pathways related to cell growth and protein synthesis were significantly underrepresented in FS (Figure 4A). These included the ribosome (*p* = 1.7e^−10^), tRNA biogenesis (*p* = 0.0014), aminoacyl‐tRNA biosynthesis (*p* = 0.0061), transcription machinery (*p* = 0.026), chaperones and folding catalysts (*p* = 0.031) and RNA polymerase (*p* = 0.043) pathways, suggesting reduced microbial protein synthesis capacity in frailty‐associated microbiomes. Notably, 26 out of the 27 KEGG functions within the cell growth pathway were all involved in different sporulation stages, and the remaining function (K06400) corresponded to a site‐specific DNA recombinase (Table S3).

Additional overrepresented pathways in the FS group (*p* = 0.01–0.001) included vitamin B6 metabolism, transporters, glycosaminoglycan degradation, glutamatergic synapse, antimicrobial resistance genes, penicillin and cephalosporin biosynthesis, lipoic acid metabolism, β‐lactam resistance, and lysosome functions (Figure 4B). In the vitamin B6 pathway, three key KEGG functions involved in *the novo* synthesis of pyridoxine, the active form of vitamin B6, were increased.

Additional metabolic pathways overrepresented in FS (*p* = 0.01–0.05) included those involved in carbohydrate metabolism, amino acid metabolism, energy production, and cellular signaling (Figure 4C). These processes are primarily related to energy acquisition and storage, the synthesis and degradation of fundamental biomolecules, and the adaptation to environmental changes.

Notably, β‐lactam resistance KEGG functions were enriched in FS, including BlaZ (K17836), MexA/B (K03585, K18138), and OMP/OprM components (K12340, K18139), all involved in multidrug efflux systems, outer membrane proteins, and multidrug resistance operons implicated in breaking down and exporting antibiotics or drugs from inside the cell to the exterior. In contrast, several functions associated with antibiotic binding proteins or entry mechanisms—for example, Opp transporters (K10823, K15581–83), BlaR1 (K02172), and PBP1a2 (K05366)—were underrepresented in FS (Figure S5). Additionally, seven KEGG functions related to antimicrobial resistance were increased in the FS group, including multidrug efflux (K18300, K18302, K18303), tetracycline inactivation (K18221), and aminoglycoside resistance (K00984, K00561).

Together, these findings suggest that the gut microbiota of older adults could employ different resistance mechanisms depending on FS status. Antibiotic resistance‐related pathways were more frequent in the FS group, while sporulation‐related KEGG functions were enriched in the NFS group.

### 3.4 Correlation Analyses

#### 3.4.1 Associations of 16S Bacterial Taxa With Functional Orthologs

**FIGURE 5:**
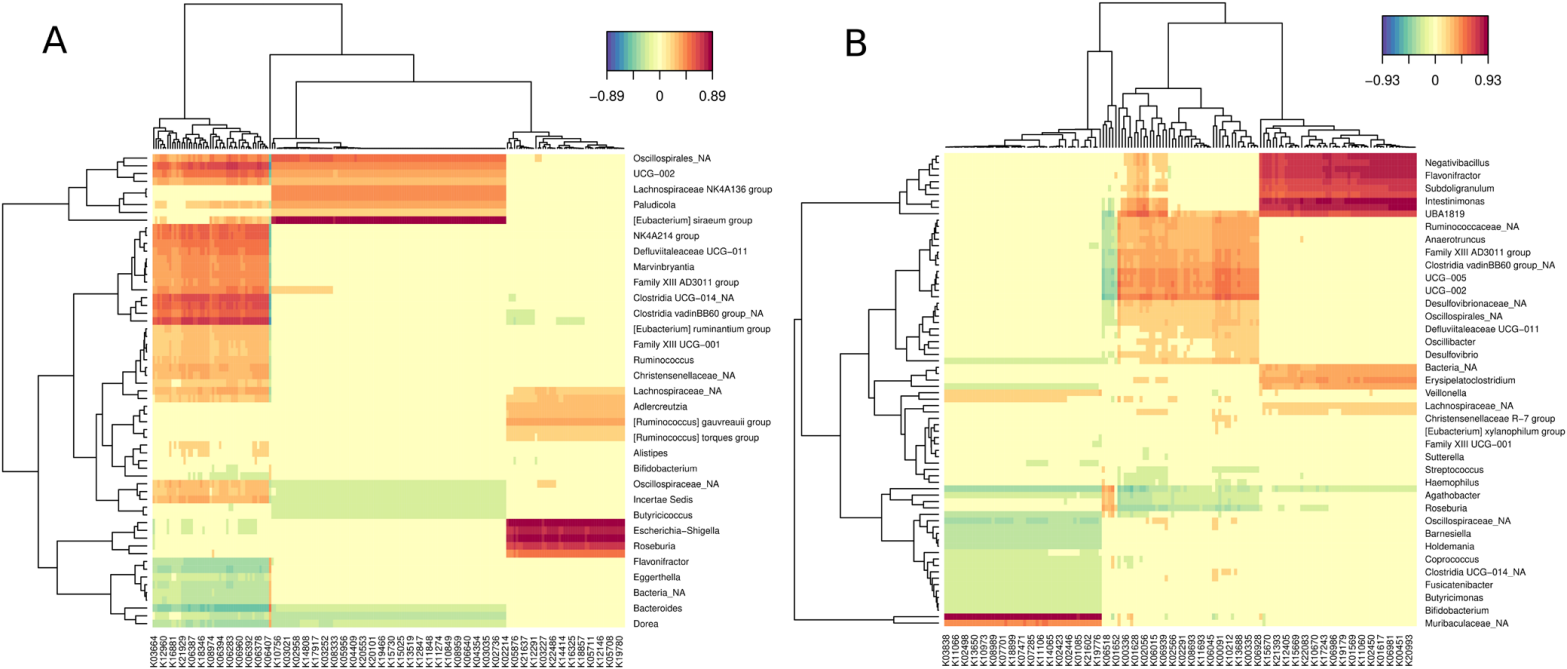
Heatmap of the correlation between genera and functional orthologs in the (A) FS group and (B) NFS group. sPLS canonical approach was made to represent the 50 variables that contributed the most to the variance in the data on each component. FS, frail; NFS, non frail.

In the FS group, strong positive correlations (*ρ* > 0.6) were observed between genera such as *Erysipelatoclostridium*, *Holdemania*, *Escherichia‐Shigella*, and *Roseburia* and KEGG functions involved in transport, metabolism, and stress pathways (Figure 5A, Table S4B), while the *Eubacterium siraeum group* showed even stronger associations (*ρ* > 0.8) with global metabolism and genetic information processing. In contrast, *Bacteroides*, *Dorea*, and *Agathobacter* were negatively correlated with these functions, suggesting divergent functional roles within the microbial community. Negative correlations were also observed between a cluster of 24 functions and *Bacteroides*, *Eggerthella*, *Agathobacter*, *Lachnoclostridium*, *Dorea*, *Blautia*, *Flavonifractor*, and *Veillonella*. In contrast, various uncertain *Clostridia* genera along with the *Christensenellaceae R‐7 group* were highly correlated (*ρ* > 0.5) with these same functions involved in nucleotide metabolism (K03784, K09769), cofactor and vitamin metabolism (K12960, K15971), genetic information processing (K21929, K01408), and transporters (K01997, K18346), among others.

In the NFS group, *Intestinimonas*, *Lachnoclostridium*, *Flavonifractor*, *Negativibacillus*, *Subdoligranulum*, *Oscillospira*, and *Ruminococcaceae UBA1819* clustered together due to the strong positive correlation with many KEGG functions involved in different metabolic pathways (Figure 5B, Table S4C). *Escherichia‐Shigella* and Muribaculaceae also correlated positively (*ρ* < 0.5) with transporters, transcription regulators, and enzymes involved in central metabolism. These same functions were negatively associated (*ρ* > −0.4) with *Faecalibacterium*, *Barnesiella*, *Holdemania*, *Alistipes*, *Erysipelotrichaceae UCG‐003*, *Fusicatenibacter*, *Butyricimonas*, *Roseburia, Coprococcus*, and *Bifidobacterium*. These patterns suggest that specific microbial taxa contribute differently to functional potential depending on frailty status, with FS‐associated bacteria linked to host–microbe interaction functions and stress‐related pathways.

#### 3.4.2 Associations of Functional Orthologs With Clinical Variables

Significant correlations (*ρ* > ±0.5) were observed between certain KEGG functions and the ILEF criteria, weight, and BMI (Figure S6, Table S4D). For the ILEF, significant positive correlations were found between the SPPB score and K00700, K06023, and K01571 (*p* = 4.5e‐5, 0.00013, 0.0002); gait speed, balance, and sit‐stand were also positively correlated with K00700 (*p* = 0.00012, 3.8e‐5, 0.006), involved in certain carbohydrates metabolism that may be linked to better physical function. Conversely, several KEGG orthologs were negatively correlated with the SPPB score and its components, including K19158 (toxin YoeB), K15725 (heavy metal efflux system), K00382 (dihydrolipoyl dehydrogenase), K04047 (DNA‐binding protein), and K17218 (sulfide: quinone oxidoreductase). Additional negative associations with functional capacity were seen for K04757 (serine/threonine‐protein kinase), K01337 (lysyl endopeptidase), K01807 (ribose 5‐phosphate isomerase A), K07118 (hypothetical protein), and K00752 (hyaluronan synthase), all of which increased in abundance as lower‐limb mobility declined. These functions might have a metabolic effect on the microbiota of these individuals.

Furthermore, negative correlations were found between BMI and weight with several KEGG functions (K14759, K12309, K06638, K14673, K04618, K08104, K17250, and K16955), suggesting that enrichment of these functions in the microbiome may be linked to healthier status. These KEGG orthologs are involved in energy production, nutrient processing, and bacterial metabolism. Notably, K14759 is a multifunctional enzyme involved in phylloquinone (vitamin K1) biosynthesis, while K12309 and K04618 are key enzymes in galactose metabolism.

### 3.5 Gut Resistome Analysis

**FIGURE 6:**
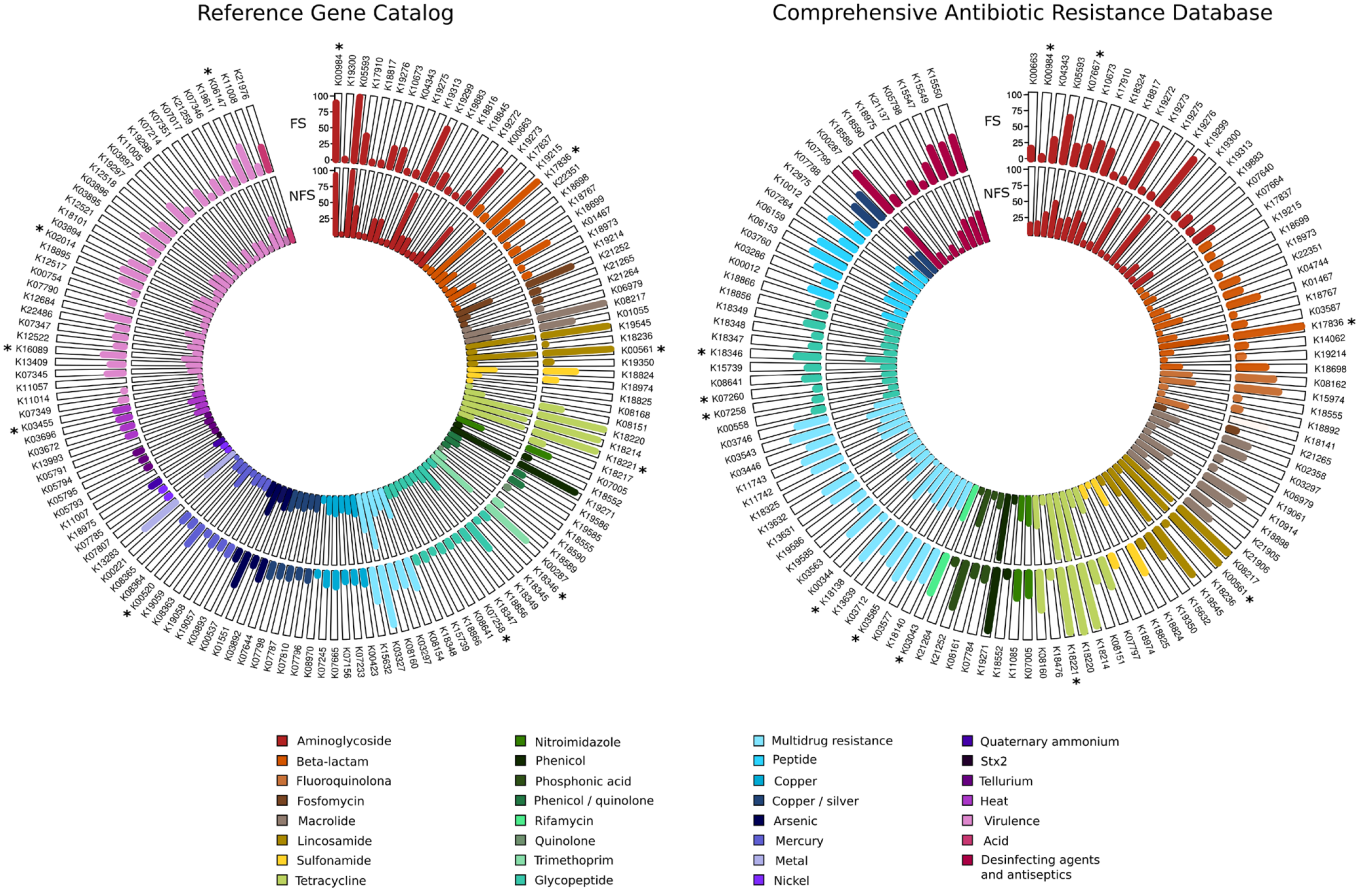
Representation of the percentage of individuals in each group (FS, NFS) with ortholog genes (KEGG functions) from two antimicrobial resistance databases. Significant differences in KEGG functions between groups are highlighted with an asterisk (*). FS, frail; NFS, non frail.

From the 10,424 KEGG functions identified in the metagenome analysis, 119 (1.14%) were associated with antimicrobial resistance (AR) based on the CARD RGI database and 140 (1.34%) according to the Reference Gene Catalog database. The FS group exhibited a 10%–15% higher abundance of reads linked to AR genes compared to the NFS group. The tetracycline resistance gene K18220 (tetM/tetO) was the most abundant across both databases, besides seven more KEGG functions present in over 90% of all individuals, potentially serving as a core reservoir for AR genes in the gut microbiota (Figure 6). These particular genes were implicated in AR to β‐lactam (K17836; β‐lactamase class A), also AR to macrolides as K00561, an erythromycin resistance methyltransferase found in many gram‐positive pathogens or the macrolide efflux protein K08217. These also included genes conferring resistance to tetracyclines (e.g., K18221, K18214), lincosamides (e.g., K19545), and chloramphenicol (e.g., K19271). Efflux and transporter‐related mechanisms dominated, followed by antibiotic inactivation and target modification.

Out of the total AR genes represented in Figure 6, 16 KEGG functions showed statistically significant differences between FS and NFS (Figure S7, Table S5). We detected in FS a high prevalence of beta‐lactam resistance KEGG functions K17836, K03585, and K18138 (*p* = 0.0013, 0.0057, 0.0075), which are part of the multidrug efflux systems and multidrug resistance operons implicated in breaking down and exporting antibiotics or drugs from the inside to the outside. Other KEGG functions that increased in the FS group were K18221 (*p* = 0.0036), a tetracycline monooxygenase; K00984 (*p* = 0.0029), the streptomycin 3″‐adenylyltransferase; K00561 (*p* = 0.011), which is an adenine dimethyltransferase; K03455 (*p* = 0.043), an H+ antiporter; and K16089 and K02014 (*p* = 0.0029, 0.035), both siderophore receptor proteins. A low prevalence of 7 KEGG functions was detected in FS: K07258 (*p* = 0.015), a penicillin‐binding protein; K18346 and K07260 (*p* = 0.0032, 0.025), responsible for vancomycin resistance; K03043 (*p* = 0.029), rifamycin‐resistant beta‐subunit of RNA polymerase; K06147 (*p* = 0.017), component of the ABC transporter system; K00520 (*p* = 0.016), a mercuric reductase; and K07667 (*p* = 0.014), involved in the regulation of the kdp operon.

## 4 Discussion

The role of gut microbiota in FS has been widely studied. However, the conclusions remain inconsistent due to variability in evaluation scales and limited consideration of comorbidities across different studies. This variability may contribute to the mixed results reported in the literature, complicating the establishment of a definitive link between gut microbiota composition and FS.

The clinical data analysis performed in this work suggests a close relationship between FS, musculoskeletal decline, and physical function, indicating that these factors may serve as complementary markers of biological aging rather than as isolated clinical outcomes.

With respect to alpha diversity analysis, our study identified a higher Chao1 index in the microbiota of frail individuals, suggesting increased species richness. However, no significant differences in the Shannon diversity index were observed between FS and NFS groups, indicating similar species evenness across groups. These findings are similar to previous studies by Almeida et al. (2022) and Xu et al. (2021), which also reported no significant differences in alpha diversity between frail and non‐frail older adults defined by Fried phenotype. Conversely, when alternative clinical measures were used to define FS, Claesson et al. (2012) demonstrated reduced species richness associated with increased functional dependence, highlighting the potential variability in gut microbiota responses to FS (Ticinesi et al. 2019). The discrepancies observed may be attributed to differences in study designs, population demographics, and FS assessment criteria, underscoring the need for standardized methodologies in future research.

We also observed compositional differences in gut microbiota associated to FS. Frail individuals had reduced Firmicutes and increased Proteobacteria, Actinobacteria, Verrucomicrobia, and Synergistetes. Similarly, Almeida et al. (2022) observed decreased Firmicutes and elevated Verrucomicrobia abundance in frail older adults. At the genus level, this study identified the enrichment of *Eggerthella*, *Christensenella*, *Oscillibacter*, *Anaerotruncus*, *Anaerofilum*, *Bacteroides*, *Parabacteroides*, *Sutterella*, and *Streptococcus* in frail individuals. These genera are consistent with microbial profiles previously linked to FS and biological aging. Specifically, the higher abundance of *Eggerthella* in FS subjects aligns with findings from Jackson et al. (2016), Picca et al. (2019), and Margiotta et al. (2020), regardless of whether FS was defined by the Rockwood index or the Fried phenotype. Several of these genera, including *Parabacteroides* and *Anaerotruncus*, have been previously linked to unhealthy aging and institutionalized older adults (Claesson et al. 2012; Ghosh et al. 2022), supporting their potential role as microbial markers of FS. In contrast, commensal bacteria like the Christensenellaceae genera, which are taxonomic markers of healthy aging, typically become more abundant with age but are depleted during unhealthy aging, as observed in the present study. We found *Erysipelotrichaceae UCG‐003*, *Christensenellaceae R‐7 group*, *Parasutterella*, *Coprococcus*, and *Hungatella* to be more abundant in the NFS group. Notably, we also saw a higher abundance of both *Christensenellaceae R‐7 group* and *Intestinibacter* in the individuals with better mobility in lower limbs (SPPB score). Studies have reported elevated levels of *Akkermansia* and *Erysipelotrichaceae UCG‐003* in the gut microbiome of healthy elders (Singh et al. 2019; Ghosh et al. 2022), and *Christensenellaceae R‐7 group* has been associated with reduced visceral adipose tissue and improved metabolic profiles in elderly populations (Tavella et al. 2021). Centenarians, who can be considered extreme examples of healthy aging, have an enrichment of Christensenellaceae in their intestinal microbiota, which promotes positive immune function, has anti‐inflammatory activity, and contributes to metabolic homeostasis (Ferrucci and Fabbri 2018).

Although physical frailty and sarcopenia are closely related conditions, both characterized by muscle impairment, no significant taxonomic or functional differences were observed in sarcopenia cases in this study. However, in our research, the SPPB score and its components were negatively correlated with six bacterial taxa, suggesting that higher abundances of these taxa are linked to poorer physical performance of the lower limbs (ILEF condition). Additionally, osteoarthritis was associated with a higher abundance of *Streptococcus*. Previous studies have reported differences in the microbiota among sarcopenic individuals (Casati et al. 2019), which may be attributed to variations in diagnostic criteria for this set of geriatric syndromes, highlighting the need for standardized approaches and consensus in their assessment.

The metagenome functional analysis revealed that biotin metabolism was the pathway with the most significant increase in the FS group. Biotin is a crucial cofactor in the catabolism of carbohydrates and proteins. High protein intake has been proposed as a strategy to mitigate FS and sarcopenia, with protein‐enriched diets shown to shift bacterial metabolism toward amino acid degradation (Beaumont et al. 2017). Additionally, other overrepresented pathways in the FS group were associated with energy production and the metabolism of alanine, aspartate, and glutamate. Aspartic acid, together with asparagine and glutamic acid, provides amino groups for the synthesis of glutamine and alanine, whose carbon skeletons can only be used for de novo synthesis of TCA cycle intermediates and glutamine. Calvani et al. (2018) reported elevated serum levels of aspartic acid, asparagine, and glutamic acid as key descriptors of the amino acid profile in older adults with FS.

Additionally, the overrepresentation of exosome‐related genes in FS individuals suggests the ability of their intestinal bacteria to interact with the host and with other bacterial cells through the release of small extracellular vesicles. Bacterial exosomes may influence the host by transporting bioactive metabolites, regulating immune response, or modulating inflammation (Ayyar and Moss 2021). Bacterial extracellular vesicles could potentially increase gut permeability and contribute to low‐grade chronic inflammation status (“inflammaging”). High levels of pro‐inflammatory markers can predict the risk of cardiovascular diseases, FS, multimorbidity, and decline of physical and cognitive function (Ferrucci and Fabbri 2018; Xu et al. 2021). Further assays will be performed in serum samples from this present cohort to determine if alterations in pro‐ and anti‐inflammatory markers are related to FS.

Another key finding of the functional analysis in our study is the potential differences in bacterial survival strategies linked to FS. Ribosomal functions were found to be overrepresented in the NFS group together with sporulation ortholog genes. These genes, associated with different stages of bacterial sporulation, included SpoII and SpoIII proteins, as well as genes responsible for the assembly of spore coats (e.g., SpoIVA and SpoV proteins). Spore‐forming bacteria are a crucial part of the human gut microbiota, primarily from the families Clostridiaceae, Bacillaceae, and Lachnospiraceae, with roles in health and disease (Egan et al. 2021). Sporulating bacteria play a role in immune modulation and gut homeostasis. They are also impacted by FS, suggesting that disruptions in spore‐forming communities might influence immune responses in older adults (Secaira‐Morocho et al. 2020). *Bacillus subtilis* has been demonstrated to promote the development of gut‐associated lymphoid tissues (GALT). Notably, this effect was found to be sporulation‐dependent, as mutants lacking the *spoA* gene were unable to induce GALT formation. It is suggested that sporulation enables *B. subtilis* cells to persist in the gut long enough to facilitate GALT development (Rhee et al. 2004). The increased presence of sporulation‐related functions in the NFS group suggests that spore‐forming bacteria are more likely to survive in the non‐frail population.

From our metagenomic analysis, we noticed a burden of AR KEGG functions present in over 90% of all individuals. Recent studies have demonstrated that the gut microbiome may act as a reservoir of AR genes, potentially to be transferred to opportunistic pathogens. Some AR genes have previously been described to be ubiquitous in all subjects regardless of age, potentially forming part of the core resistome (Anthony et al. 2021). Interestingly, aging was found to be associated with a higher burden of AR, especially genes encoding multidrug efflux pumps (Tavella et al. 2021; Lu et al. 2014). Similar results were found in this study since antibiotic efflux and transporters were the dominant mechanisms in our cohort. We also see in this study that AR genetic information is 10%–15% higher in frail individuals, similar to a previous study in which elderly people from nursing homes had an increased number, abundance, and diversity of antimicrobial resistance genes compared to healthy subjects (Araos et al. 2019). A recent review on antibiotic resistance in the elderly by Theodorakis et al. (2024) highlights that prolonged immobility can result in the development of ulcers and chronic wounds, which act as entry points for resistant pathogens.

Additionally, malnutrition and chronic low‐grade inflammation associated with FS further contribute to the persistence and spread of antibiotic‐resistant pathogens (Theodorakis et al. 2024). The most prevalent KEGG functions we obtained in the FS group were related to tetracycline, followed by aminoglycoside, β‐lactam, and macrolide resistance. Wu et al. (2021) described that tetracycline, macrolide–lincosamide–streptogramin (MLS), aminoglycoside, and sulfonamide and sulfone (SS) were the top five AR gene types in each of the age groups, with tetracycline being the most abundant in elders. Moreover, our results suggest that β‐lactam resistance mechanisms are different between FS and NFS groups; overrepresented functions in FS are linked to β‐lactam degradation and export outside the bacteria, whereas underrepresented functions are associated with antibiotic entry mechanisms. No previous literature has been found about these different mechanisms in the gut bacteria. Still, Theodorakis et al. (2024) described that an extended‐spectrum beta‐lactamase‐producing Enterobacteriaceae had the highest median prevalence in the elderly. The importance of antibiotic‐resistant gut bacteria as an immediate and long‐term threat to human health is well established, particularly in vulnerable groups such as elderly individuals with a debilitated immune system. However, there is limited information on how the human intestinal resistome changes during unhealthy aging.

In the correlation analysis between taxonomic composition and functional data in the NFS group, weak negative correlations were observed between certain KEGG functions and butyrate‐producing bacteria, including genera such as *Faecalibacterium*, *Butyricimonas*, *Roseburia*, *Coprococcus*, and Clostridiaceae genera. Numerous functions were identified, primarily related to transporter proteins, transcription factors, and enzymes involved in global metabolism, among others. Since butyrate‐producing bacteria are widely associated with healthy aging (Claesson et al. 2012; Gosh et al. 2020; Araújo et al. 2024), further metatranscriptomic analysis could help determine whether these functions are actively transcribed and explore their potential implications in these healthy individuals.

This study highlights the complex interplay between gut microbiota, functional frailty, and aging, emphasizing the need for integrative approaches to address variability in current findings. Our results provide important insights into the microbial profiles associated with FS, particularly the enrichment of potential pathogenic taxa and depletion of beneficial commensals in frail individuals. Additionally, functional analyses revealed key metabolic pathways and resistance mechanisms that could influence FS progression and host health. By identifying specific microbiota taxa and functions linked to FS, this work contributes to future investigations for a better understanding of the gut bacteria's role in aging‐related conditions. Additional covariates in microbiome analyses enhance the biological relevance of associations and help to improve targeted interventions in older adults.

Although this study offers new insights, some limitations were found. Detailed information on diet and medication would help to better understand host–microbiome interactions. Additionally, a more balanced distribution of frail men and women might strengthen sex‐related analyses, and including rural populations could make the findings apply more broadly. Furthermore, because covariate adjustment was limited to sensitivity analyses, residual confounding may remain, suggesting that future studies should examine these associations in detail. To our knowledge, this is the first study that has specifically investigated the profile of microbiome antimicrobial resistance in elderly populations with geriatric syndromes such as FS. Furthermore, given the ecological and functional significance of spore‐forming bacteria, a comprehensive analysis of the resistome alongside the sporobiota in the gut of older individuals could offer valuable insights into their interactions and combined roles in host health and microbial resilience.

## Author Contributions

Ana Barberá and Susana Ruiz‐Ruiz carried out the experiments, processed sequencing data, and conducted the statistical analyses. Rosario Ortolá, Mercedes Sotos‐Prieto, and Fernando Rodríguez‐Artalejo contributed to samples and data collection and clinical database generation. Ana Barberá, Susana Ruiz‐Ruiz, and Andrés Moya wrote the manuscript. Susana Ruiz‐Ruiz, Andrés Moya, and Fernando Rodríguez‐Artalejo designed the study. All authors contributed to the article and approved the submitted version.

## Funding

This research was funded by the Spanish Ministry of Science, Innovation and Universities (project number PID2019‐105969GB‐I00 funding by MICIU/AEI/https://doi.org/10.13039/501100011033), by the Carlos III Health Institute (ISCIII) (project number PMPTA22/00107, PMPTA22/00037 and PMPTA23/00001), and supported by INVEST/2022/309 grant from the Next Generation‐EU plan and FIS grant 22/1111 from ISCIII; the Secretary of *R* + D + I; and the ERDF/ESF.

## Conflicts of Interest

The authors declare no conflicts of interest.

## Supporting information


**Figure S1:** Boxplots for the alpha diversity Chao1 and Shannon indexes comparing FS and NFS (A) and all groups (B, C): frailty and non‐frailty females (FS.F, NFS.F) and frailty and non‐frailty males (FS.M, NFS.M). FS, frail; NFS, non frail.

**Figure S2:** PCoA plots and Adonis test of the 16S taxonomic differences between groups for each categorical clinical feature.

**Figure S3:** PCoA plots and Adonis test of the functional differences between groups for each categorical clinical feature using KEGG functions data from shotgun sequencing.

**Figure S4:** Principal Coordinates Analysis (PCoA) based on Bray‐Curtis distances for (A) FS versus NFS, (B) frail, and (C) non‐frail females and males for KEGG functions.

**Figure S5:** β‐lactam resistance pathway. Overrepresented KEGG functions in the frail (FS) group are colored blue, while underrepresented functions in the FS group are colored red. Taken from https://www.genome.jp/kegg and rendered by Pathview (Luo and Brouwer 2013).

**Figure S6:** Network map of the correlations between the KEGG functions and continuous clinical data. Spearman coefficient (*ρ*) equal to or greater than ±0.5 was used to plot the connections.

**Figure S7:** Boxplots of the statistically significant KEGG functions involved in AR for the DESeq2 test between FS and NFS groups from both CARD and Reference Gene Catalog databases. FS, frail; NFS, non frail.

**Table S1:** Linear mixed effect model from the 16S taxonomy alpha diversity using the model matrix FS + variable.

**Table S2:** 16S taxonomic analysis between groups at the phylum level.

**Table S3:** Sporulation‐related KEGG functions underrepresented in FS.

**Table S4:** Correlation analysis between 16S characterized genera and continuous clinical information performed by mixOmics sPLS‐canonical.

**Table S5:** Differential KEGG functions between FS and NFS in the resistome analysis.

## Data Availability

The datasets presented in this study are deposited in the European Nucleotide Archive repository, https://www.ebi.ac.uk/ena/browser/view/PRJEB83322. The figures and data on which the present work is based can be found at Figshare: https://doi.org/10.6084/m9.figshare.28658663.

## References

[acel70365-bib-0001] Alcock, B. P. , W. Huynh , R. Chalil , et al. 2023. “CARD 2023: Expanded Curation, Support for Machine Learning, and Resistome Prediction at the Comprehensive Antibiotic Resistance Database.” Nucleic Acids Research 51, no. D1: D690–D699. 10.1093/nar/gkac920. 36263822 PMC9825576

[acel70365-bib-0002] Almeida, H. M. , A. V. Sardeli , J. Conway , N. A. Duggal , and C. R. Cavaglieri . 2022. “Comparison Between Frail and Non‐Frail Older Adults' Gut Microbiota: A Systematic Review and Meta‐Analysis.” Ageing Research Reviews 82: 101773. 10.1016/j.arr.2022.101773. 36349647

[acel70365-bib-0003] Anthony, W. E. , C. D. Burnham , G. Dantas , and J. H. Kwon . 2021. “The Gut Microbiome as a Reservoir for Antimicrobial Resistance.” Journal of Infectious Diseases 223, no. Suppl 2: S209–S213. 10.1093/infdis/jiaa497. 33326581 PMC8206794

[acel70365-bib-0004] Araos, R. , T. Battaglia , J. A. Ugalde , M. Rojas‐Herrera , M. J. Blaser , and E. M. C. D'Agata . 2019. “Fecal Microbiome Characteristics and the Resistome Associated With Acquisition of Multidrug‐Resistant Organisms Among Elderly Subjects.” Frontiers in Microbiology 10: 2260. 10.3389/fmicb.2019.02260. 31611867 PMC6777474

[acel70365-bib-0005] Araújo, J. R. , C. Marques , C. Rodrigues , C. Calhau , and A. Faria . 2024. “The Metabolic and Endocrine Impact of Diet‐Derived Gut Microbiota Metabolites on Ageing and Longevity.” Ageing Research Reviews 100: 102451. 10.1016/j.arr.2024.102451. 39127442

[acel70365-bib-0006] Ayyar, K. K. , and A. C. Moss . 2021. “Exosomes in Intestinal Inflammation.” Frontiers in Pharmacology 12: 658505. 10.3389/fphar.2021.658505. 34177577 PMC8220320

[acel70365-bib-0056] Badal, V. D. , E. D. Vaccariello , E. R. Murray , et al. 2020. “The Gut Microbiome, Aging, and Longevity: A Systematic Review.” Nutrients 12, no. 12: 3759. 10.3390/nu12123759. 33297486 PMC7762384

[acel70365-bib-0007] Beaumont, M. , K. J. Portune , N. Steuer , et al. 2017. “Quantity and Source of Dietary Protein Influence Metabolite Production by Gut Microbiota and Rectal Mucosa Gene Expression: A Randomized, Parallel, Double‐Blind Trial in Overweight Humans.” American Journal of Clinical Nutrition 106, no. 4: 1005–1019. 10.3945/ajcn.117.158816. 28903954

[acel70365-bib-0008] Callahan, B. J. , P. J. McMurdie , M. J. Rosen , A. W. Han , A. J. A. Johnson , and S. P. Holmes . 2016. “DADA2: High‐Resolution Sample Inference From Illumina Amplicon Data.” Nature Methods 13, no. 7: 581–583. 10.1038/nmeth.3869. 27214047 PMC4927377

[acel70365-bib-0009] Calvani, R. , A. Picca , F. Marini , et al. 2018. “A Distinct Pattern of Circulating Amino Acids Characterizes Older Persons With Physical Frailty and Sarcopenia: Results From the BIOSPHERE Study.” Nutrients 10, no. 11: 1691. 10.3390/nu10111691. 30404172 PMC6265849

[acel70365-bib-0010] Casati, M. , E. Ferri , D. Azzolino , M. Cesari , and B. Arosio . 2019. “Gut Microbiota and Physical Frailty Through the Mediation of Sarcopenia.” Experimental Gerontology 124: 110639. 10.1016/j.exger.2019.110639. 31226349

[acel70365-bib-0011] Chen, S. , Y. Zhou , Y. Chen , and J. Gu . 2018. “fastp: An ultra‐fast all‐in‐one FASTQ preprocessor.” Bioinformatics 34, no. 17: i884–i890. 10.1093/bioinformatics/bty560. 30423086 PMC6129281

[acel70365-bib-0012] Claesson, M. J. , I. B. Jeffery , S. Conde , et al. 2012. “Gut Microbiota Composition Correlates With Diet and Health in the Elderly.” Nature 488, no. 7410: 178–184. 10.1038/nature11319. 22797518

[acel70365-bib-0013] Cruz‐Jentoft, A. J. , G. Bahat , J. Bauer , et al. 2019. “Sarcopenia: Revised European Consensus on Definition and Diagnosis.” Age and Ageing 48, no. 4: 601. 10.1093/ageing/afz046. PMC6593317 31081853

[acel70365-bib-0014] Davinelli, S. , G. Corbi , and G. Scapagnini . 2021. “Frailty Syndrome: A Target for Functional Nutrients?” Mechanisms of Ageing and Development 195: 111441. 10.1016/j.mad.2021.111441. 33539905

[acel70365-bib-0015] Dent, E. , F. C. Martin , H. Bergman , J. Woo , R. Romero‐Ortuno , and J. D. Walston . 2019. “Management of Frailty: Opportunities, Challenges, and Future Directions.” Lancet 394, no. 10206: 1376–1386. 10.1016/S0140-6736(19)31785-4. 31609229

[acel70365-bib-0016] Egan, M. , E. Dempsey , C. A. Ryan , R. P. Ross , and C. Stanton . 2021. “The Sporobiota of the Human Gut.” Gut Microbes 13, no. 1: 1–17. 10.1080/19490976.2020.1863134. PMC7801112 33406976

[acel70365-bib-0017] Feldgarden, M. , V. Brover , N. Gonzalez‐Escalona , et al. 2021. “AMRFinderPlus and the Reference Gene Catalog Facilitate Examination of the Genomic Links Among Antimicrobial Resistance, Stress Response, and Virulence.” Scientific Reports 11, no. 1: 12728. 10.1038/s41598-021-91456-0. 34135355 PMC8208984

[acel70365-bib-0018] Ferrucci, L. , and E. Fabbri . 2018. “Inflammageing: Chronic Inflammation in Ageing, Cardiovascular Disease, and Frailty.” Nature Reviews Cardiology 15, no. 9: 505–522. 10.1038/s41569-018-0064-2. 30065258 PMC6146930

[acel70365-bib-0019] Fried, L. P. , C. M. Tangen , J. Walston , et al. 2001. “Frailty in Older Adults: Evidence for a Phenotype.” Journals of Gerontology Series A: Biological Sciences and Medical Sciences 56, no. 3: M146–M156. 10.1093/gerona/56.3.m146. 11253156

[acel70365-bib-0020] García‐Esquinas, E. , R. Ortolá , D. Martínez‐Gómez , et al. 2021. “Causal Effects of Physical Activity and Sedentary Behaviour on Health Deficits Accumulation in Older Adults.” International Journal of Epidemiology 50, no. 3: 852–865. 10.1093/ije/dyaa228. 33150410

[acel70365-bib-0021] García‐García, F. J. , G. Gutierrez , A. Alfaro‐Acha , et al. 2011. “The Prevalence of Frailty Syndrome in an Older Population From Spain. The Toledo Study for Healthy Aging.” Journal of Nutrition, Health & Aging 15, no. 10: 852–856. 10.1007/s12603-011-0075-8.22159772

[acel70365-bib-0057] Ghosh, T. S. , S. Rampelli , I. B. Jeffery , et al. 2020. “Mediterranean Diet Intervention Alters the Gut Microbiome in Older People Reducing Frailty and Improving Health Status: The NU‐AGE 1‐Year Dietary Intervention Across Five European Countries.” Gut 69, no. 7: 1218. 10.1136/gutjnl-2019-319654. 32066625 PMC7306987

[acel70365-bib-0022] Ghosh, T. S. , F. Shanahan , and P. W. O'Toole . 2022. “The Gut Microbiome as a Modulator of Healthy Ageing.” Nature Reviews Gastroenterology & Hepatology 19, no. 9: 565–584. 10.1038/s41575-022-00605-x. 35468952 PMC9035980

[acel70365-bib-0023] Guo, Y. , G. Zhu , F. Wang , et al. 2022. “Distinct Serum and Fecal Metabolite Profiles Linking With Gut Microbiome in Older Adults With Frailty.” Frontiers in Medicine 9: 827174. 10.3389/fmed.2022.827174. 35479954 PMC9035822

[acel70365-bib-0024] Guralnik, J. M. , E. M. Simonsick , L. Ferrucci , et al. 1994. “A Short Physical Performance Battery Assessing Lower Extremity Function: Association With Self‐Reported Disability and Prediction of Mortality and Nursing Home Admission.” Journal of Gerontology 49, no. 2: M85–M94. 10.1093/geronj/49.2.m85. 8126356

[acel70365-bib-0025] Jackson, M. A. , I. B. Jeffery , M. Beaumont , et al. 2016. “Signatures of Early Frailty in the Gut Microbiota.” Genome Medicine 8, no. 1: 21. 10.1186/s13073-016-0275-2. 26822992 PMC4731918

[acel70365-bib-0026] Khan, K. T. , K. Hemati , and A. L. Donovan . 2019. “Geriatric Physiology and the Frailty Syndrome.” Anesthesiology Clinics 37, no. 3: 453–474. 10.1016/j.anclin.2019.04.006. 31337478

[acel70365-bib-0027] Kursa, M. B. , and W. R. Rudnicki . 2010. “Feature Selection With the Boruta Package.” Journal of Statistical Software 36, no. 11: 1–13. https://www.jstatsoft.org/index.php/jss/article/view/v036i11.

[acel70365-bib-0058] Langmead, B. , and S. L. Salzberg . 2012. “Fast Gapped‐Read Alignment With Bowtie 2.” Nature Methods 9, no. 4: 357–359. 10.1038/nmeth.1923. 22388286 PMC3322381

[acel70365-bib-0028] Lê Cao, K. A. , S. Boitard , and P. Besse . 2011. “Sparse PLS Discriminant Analysis: Biologically Relevant Feature Selection and Graphical Displays for Multiclass Problems.” BMC Bioinformatics 12: 253. 10.1186/1471-2105-12-253. 21693065 PMC3133555

[acel70365-bib-0029] Lin, H. , and S. D. Peddada . 2020. “Analysis of Compositions of Microbiomes With Bias Correction.” Nature Communications 11, no. 1: 3514. 10.1038/s41467-020-17041-7. PMC7360769 32665548

[acel70365-bib-0030] Lu, N. , Y. Hu , L. Zhu , et al. 2014. “DNA Microarray Analysis Reveals That Antibiotic Resistance‐Gene Diversity in Human Gut Microbiota Is Age Related.” Scientific Reports 4: 4302. 10.1038/srep04302. 24618772 PMC3950639

[acel70365-bib-0031] Luo, W. , and C. Brouwer . 2013. “Pathview: An R/Bioconductor Package for Pathway‐Based Data Integration and Visualization.” Bioinformatics 29, no. 14: 1830–1831. 10.1093/bioinformatics/btt285. 23740750 PMC3702256

[acel70365-bib-0032] Margiotta, E. , F. Miragoli , M. L. Callegari , et al. 2020. “Gut Microbiota Composition and Frailty in Elderly Patients With Chronic Kidney Disease.” PLoS One 15, no. 4: e0228530. 10.1371/journal.pone.0228530. 32236095 PMC7112193

[acel70365-bib-0033] Menzel, P. , K. L. Ng , and A. Krogh . 2016. “Fast and Sensitive Taxonomic Classification for Metagenomics With Kaiju.” Nature Communications 7: 11257. 10.1038/ncomms11257. PMC4833860 27071849

[acel70365-bib-0034] Mitnitski, A. B. , A. J. Mogilner , and K. Rockwood . 2001. “Accumulation of Deficits as a Proxy Measure of Aging.” Scientific World Journal 1: 323–336. 10.1100/tsw.2001.58. 12806071 PMC6084020

[acel70365-bib-0035] O'Caoimh, R. , L. Galluzzo , Á. Rodríguez‐Laso , et al. 2018. “Prevalence of Frailty at Population Level in European ADVANTAGE Joint Action Member States: A Systematic Review and Meta‐Analysis.” Annali dell'Istituto Superiore di Sanità 54, no. 3: 226–238. 10.4415/ANN_18_03_10. 30284550

[acel70365-bib-0036] Ortolá, R. , E. García‐Esquinas , M. Sotos‐Prieto , et al. 2022. “Mediterranean Diet and Changes in Frequency, Severity, and Localization of Pain in Older Adults: The Seniors‐ENRICA Cohorts.” Journals of Gerontology, Series A: Biological Sciences and Medical Sciences 77, no. 1: 122–130. 10.1093/gerona/glab109. 33839765

[acel70365-bib-0037] O'Toole, P. W. , and I. B. Jeffery . 2015. “Gut Microbiota and Aging.” Science 350, no. 6265: 1214–1215. 10.1126/science.aac8469. 26785481

[acel70365-bib-0038] Ottenbacher, K. J. , L. G. Branch , L. Ray , V. A. Gonzales , M. K. Peek , and M. R. Hinman . 2002. “The Reliability of Upper‐ and Lower‐Extremity Strength Testing in a Community Survey of Older Adults.” Archives of Physical Medicine and Rehabilitation 83, no. 10: 1423–1427. 10.1053/apmr.2002.34619. 12370879

[acel70365-bib-0039] Picca, A. , F. R. Ponziani , R. Calvani , et al. 2019. “Gut Microbial, Inflammatory and Metabolic Signatures in Older People With Physical Frailty and Sarcopenia: Results From the BIOSPHERE Study.” Nutrients 12, no. 1: 65. 10.3390/nu12010065. 31887978 PMC7019826

[acel70365-bib-0040] Ragonnaud, E. , and A. Biragyn . 2021. “Gut Microbiota as the Key Controllers of “Healthy” Aging of Elderly People.” Immunity & Ageing 18, no. 1: 2. 10.1186/s12979-020-00213-w. 33397404 PMC7784378

[acel70365-bib-0041] Rhee, K. J. , P. Sethupathi , A. Driks , D. K. Lanning , and K. L. Knight . 2004. “Role of Commensal Bacteria in Development of Gut‐Associated Lymphoid Tissues and Preimmune Antibody Repertoire.” Journal of Immunology 172, no. 2: 1118–1124. 10.4049/jimmunol.172.2.1118. 14707086

[acel70365-bib-0042] Rodríguez‐Mañas, L. , C. Féart , G. Mann , et al. 2013. “Searching for an Operational Definition of Frailty: A Delphi Method‐Based Consensus Statement: The Frailty Operative Definition‐Consensus Conference Project.” Journals of Gerontology, Series A: Biological Sciences and Medical Sciences 68, no. 1: 62–67. 10.1093/gerona/gls119. 22511289 PMC3598366

[acel70365-bib-0043] Ruiz‐Grosso, P. , C. Loret de Mola , J. M. Vega‐Dienstmaier , et al. 2012. “Validation of the Spanish Center for Epidemiological Studies Depression and Zung Self‐Rating Depression Scales: A Comparative Validation Study.” PLoS One 7, no. 10: e45413. 10.1371/journal.pone.0045413. 23056202 PMC3466285

[acel70365-bib-0044] Sánchez, B. , B. E. Martínez , J. F. Aguirre , et al. 2022. “Emerging Evidence on the Use of Probiotics and Prebiotics to Improve the Gut Microbiota of Older Adults With Frailty Syndrome: A Narrative Review.” Journal of Nutrition, Health & Aging 26, no. 10: 926–935. 10.1007/s12603-022-1842-4. PMC9483424 36259581

[acel70365-bib-0045] Schoultz, I. , M. J. Claesson , M. G. Dominguez‐Bello , et al. 2025. “Gut Microbiota Development Across the Lifespan: Disease Links and Health‐Promoting Interventions.” Journal of Internal Medicine 297, no. 6: 560–583. 10.1111/joim.20089. 40270478 PMC12087861

[acel70365-bib-0046] Secaira‐Morocho, H. , J. A. Castillo , and A. Driks . 2020. “Diversity and Evolutionary Dynamics of Spore‐Coat Proteins in Spore‐Forming Species of Bacillales.” Microbial Genomics 6, no. 11: mgen000451. 10.1099/mgen.0.000451. 33052805 PMC7725329

[acel70365-bib-0047] Singh, H. , M. G. Torralba , K. J. Moncera , et al. 2019. “Gastro‐Intestinal and Oral Microbiome Signatures Associated With Healthy Aging.” Geroscience 41, no. 6: 907–921. 10.1007/s11357-019-00098-8. 31620923 PMC6925087

[acel70365-bib-0048] Tamames, J. , and F. Puente‐Sánchez . 2019. “SqueezeMeta, a Highly Portable, Fully Automatic Metagenomic Analysis Pipeline.” Frontiers in Microbiology 9: 3349. 10.3389/fmicb.2018.03349. 30733714 PMC6353838

[acel70365-bib-0049] Tavella, T. , S. Turroni , P. Brigidi , M. Candela , and S. Rampelli . 2021. “The Human Gut Resistome up to Extreme Longevity.” mSphere 6, no. 5: e0069121. 10.1128/mSphere.00691-21. 34494880 PMC8550338

[acel70365-bib-0050] Theodorakis, N. , G. Feretzakis , C. Hitas , et al. 2024. “Antibiotic Resistance in the Elderly: Mechanisms, Risk Factors, and Solutions.” Microorganisms 12, no. 10: 1978. 10.3390/microorganisms12101978. 39458286 PMC11509523

[acel70365-bib-0051] Ticinesi, A. , A. Nouvenne , N. Cerundolo , et al. 2019. “Gut Microbiota, Muscle Mass and Function in Aging: A Focus on Physical Frailty and Sarcopenia.” Nutrients 11, no. 7: 1633. 10.3390/nu11071633. 31319564 PMC6683074

[acel70365-bib-0052] van Tongeren, S. P. , J. P. Slaets , H. J. Harmsen , and G. W. Welling . 2005. “Fecal Microbiota Composition and Frailty.” Applied and Environmental Microbiology 71, no. 10: 6438–6442. 10.1128/AEM.71.10.6438-6442.2005. 16204576 PMC1265947

[acel70365-bib-0053] Wu, L. , X. Xie , Y. Li , et al. 2021. “Metagenomics‐Based Analysis of the Age‐Related Cumulative Effect of Antibiotic Resistance Genes in Gut Microbiota.” Antibiotics 10, no. 8: 1006. 10.3390/antibiotics10081006. 34439056 PMC8388928

[acel70365-bib-0054] Xu, Y. , Y. Wang , H. Li , et al. 2021. “Altered Fecal Microbiota Composition in Older Adults With Frailty.” Frontiers in Cellular and Infection Microbiology 11: 696186. 10.3389/fcimb.2021.696186. 34485176 PMC8415883

[acel70365-bib-0055] Ye, H. , T. S. Ghosh , C. M. Hueston , et al. 2023. “Engraftment of Aging‐Related Human Gut Microbiota and the Effect of a Seven‐Species Consortium in a Pre‐Clinical Model.” Gut Microbes 15, no. 2: 2282796. 10.1080/19490976.2023.2282796. 38010168 PMC10854441

