# Supplementary figures and images for: The Role of the Gut Microbiome in the Complex Network of Frailty Syndrome and Associated Comorbidities in Aging
Source: Aging Cell. 2026 Jan 10;25(2):e70365. doi: 10.1111/acel.70365 (PMC12790095; doi:10.1111/acel.70365)

**A****Chao1**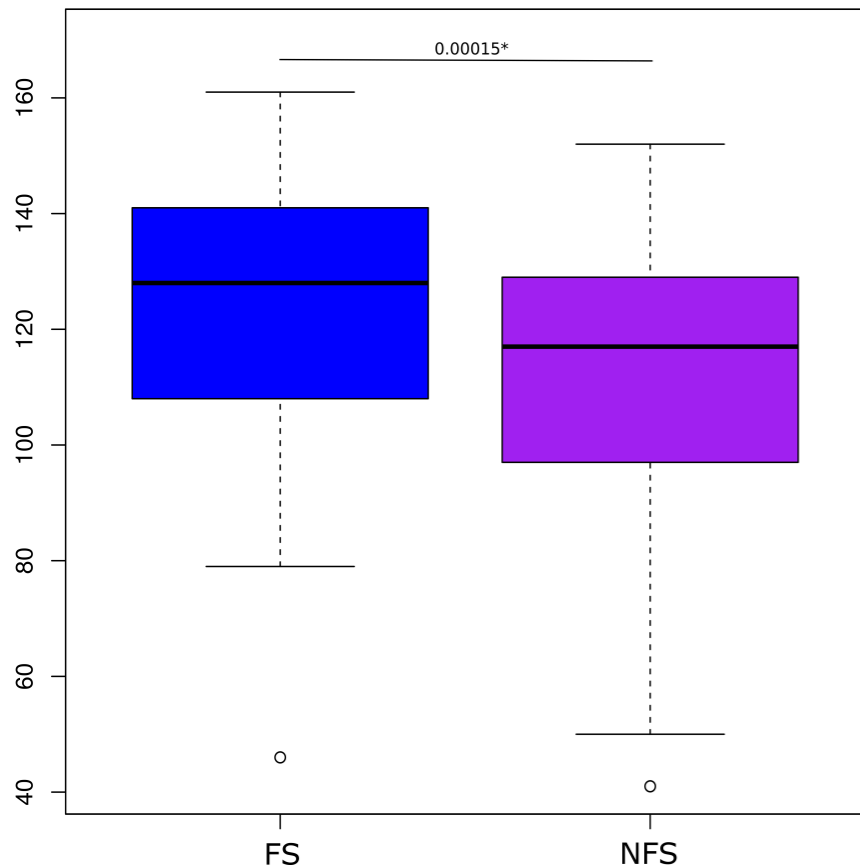**B****Chao1**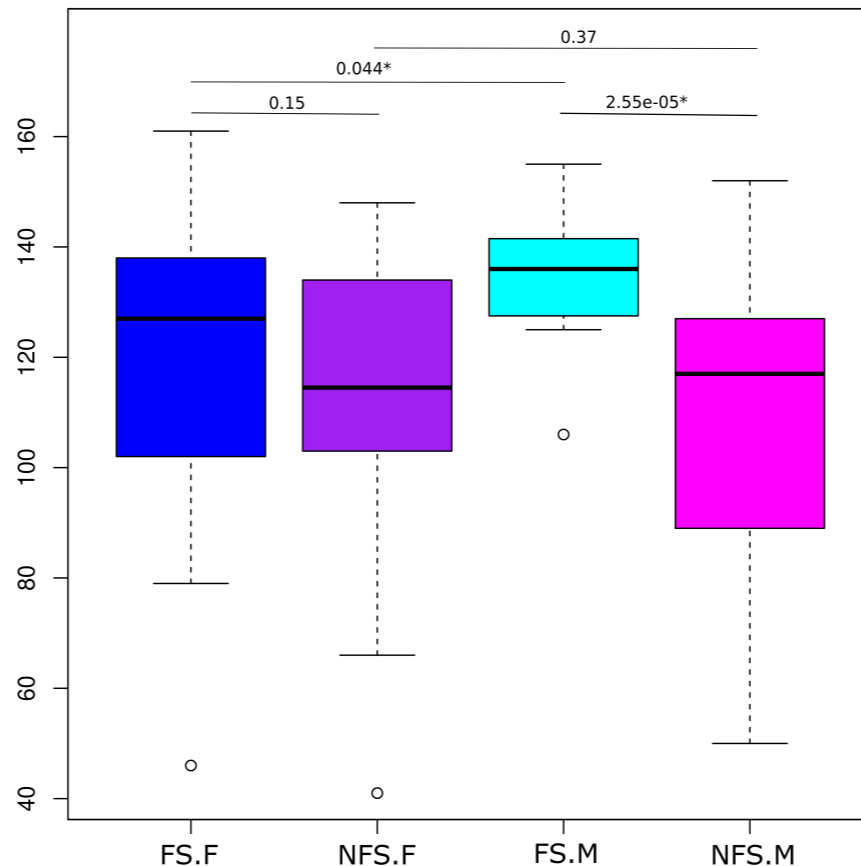**C****Shannon**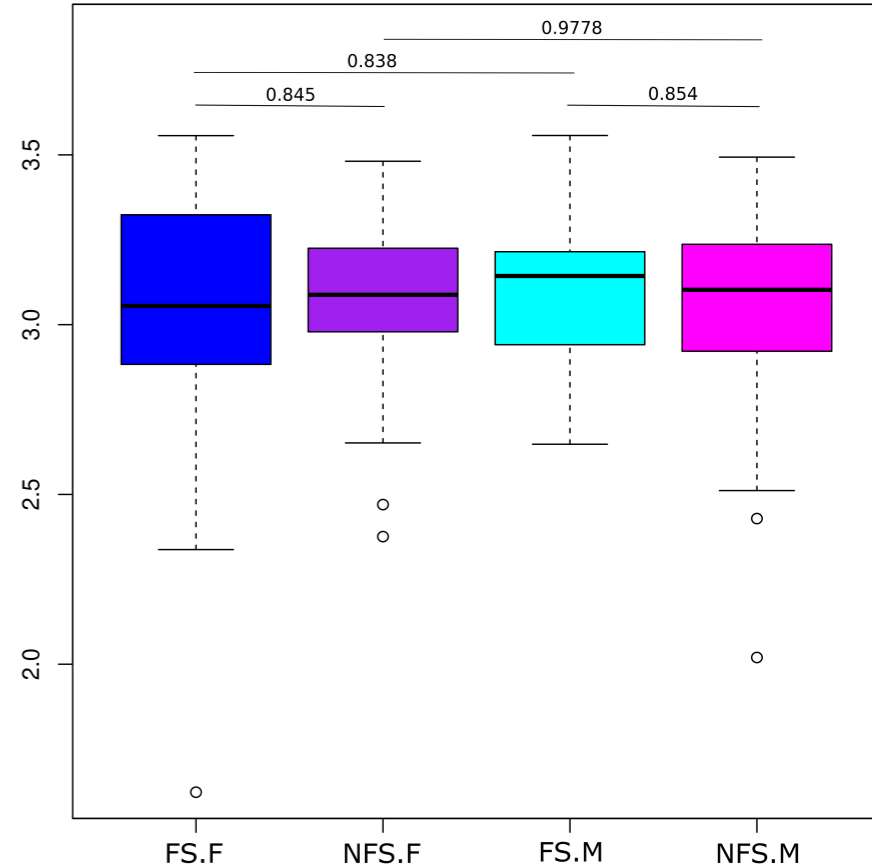

Supplement: Supplementary file 1 — Figure S1: Boxplots for the alpha diversity Chao1 and Shannon indexes comparing FS and NFS (A) and all groups (B, C): frailty and non‐frailty females (FS.F, NFS.F) and frailty and non‐frailty males (FS.M, NFS.M). FS, frail; NFS, non frail. [file ACEL-25-e70365-s007.pdf]

A

permanova p-value: 0.00166

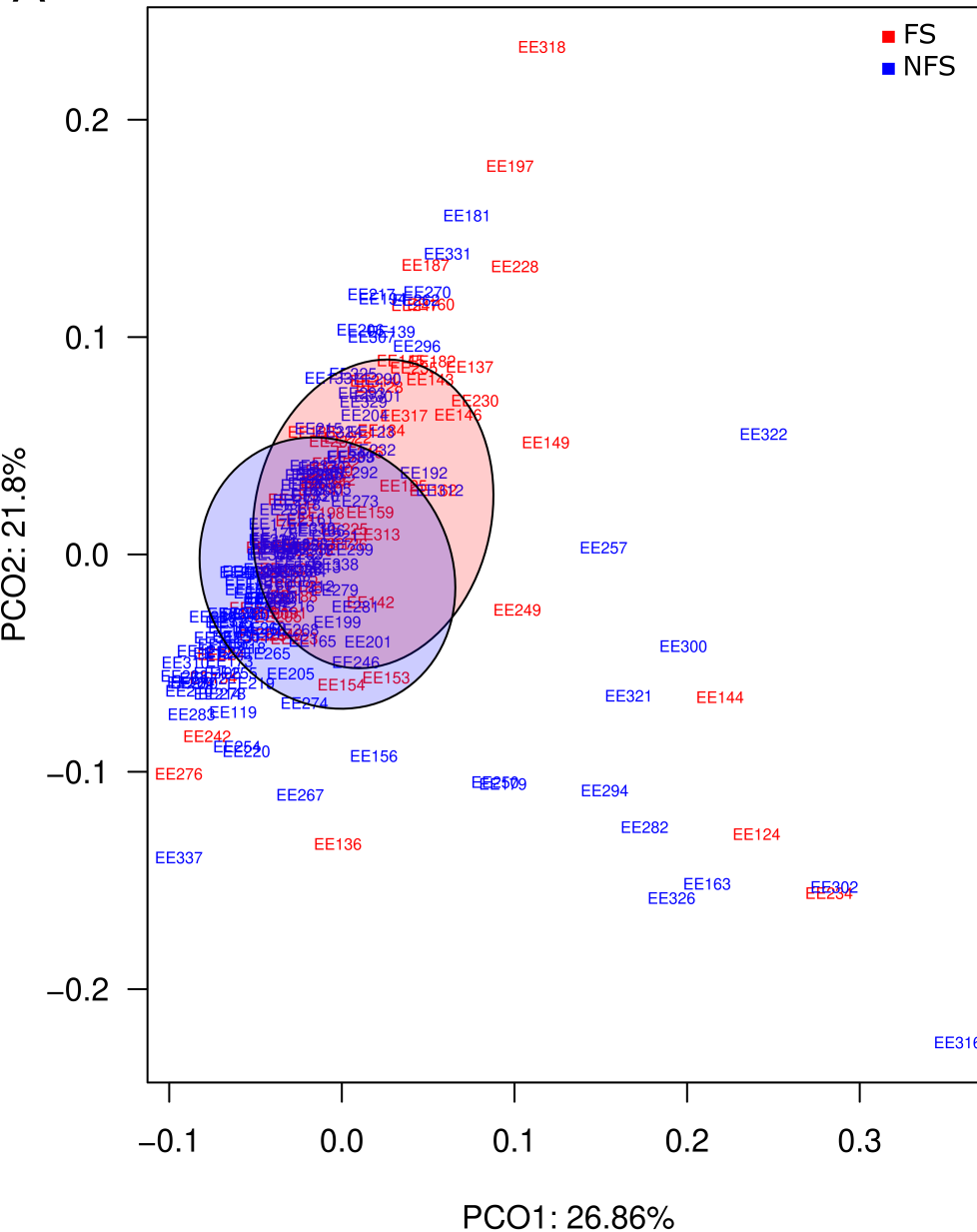

B

permanova p-value: 0.0366

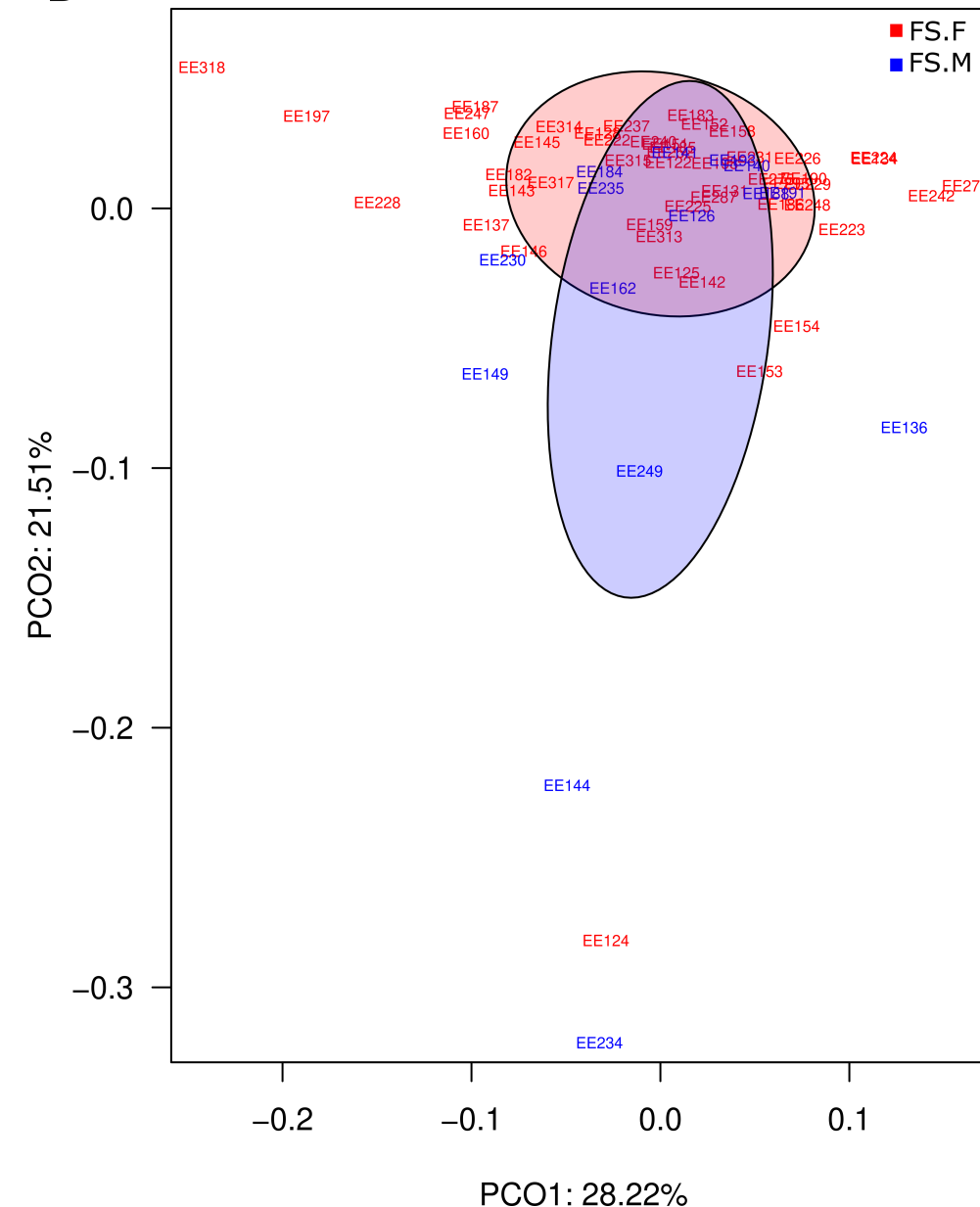

C

permanova p-value: 0.215

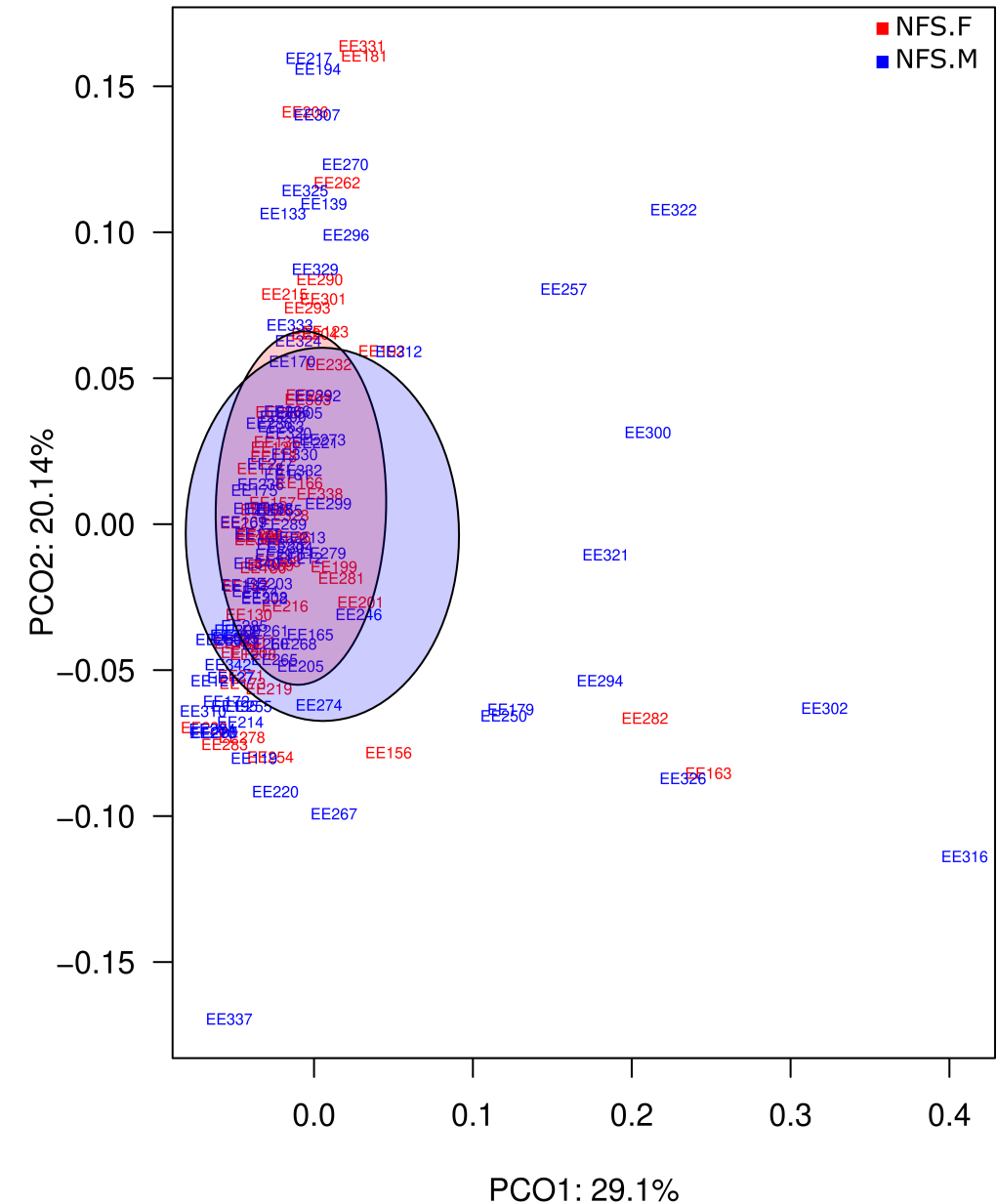

Supplement: Supplementary file 4 — Figure S4: Principal Coordinates Analysis (PCoA) based on Bray‐Curtis distances for (A) FS versus NFS, (B) frail, and (C) non‐frail females and males for KEGG functions. [file ACEL-25-e70365-s010.pdf]

# beta-LACTAM RESISTANCE

■ Underrepresented  
■ Overrepresented

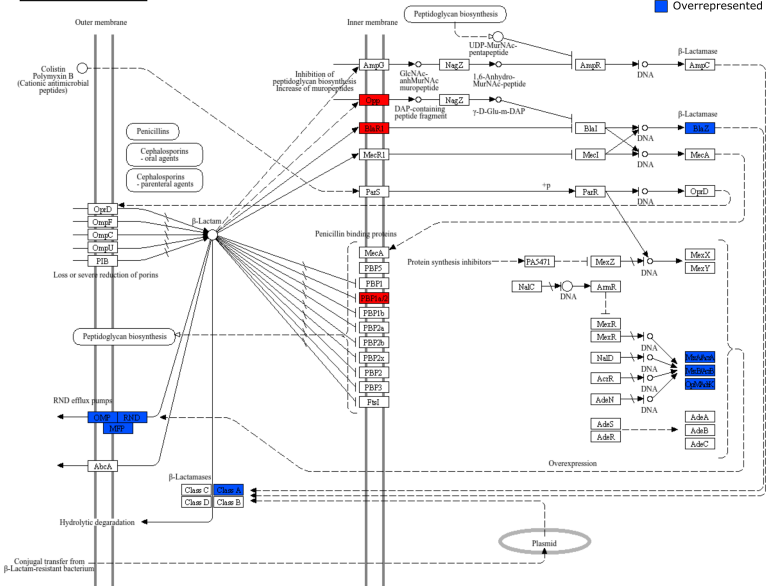

Supplement: Supplementary file 5 — Figure S5: β‐lactam resistance pathway. Overrepresented KEGG functions in the frail (FS) group are colored blue, while underrepresented functions in the FS group are colored red. Taken from https://www.genome.jp/kegg and rendered by Pathview (Luo and Brouwer 2013). [file ACEL-25-e70365-s002.pdf]

Color key

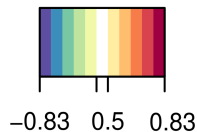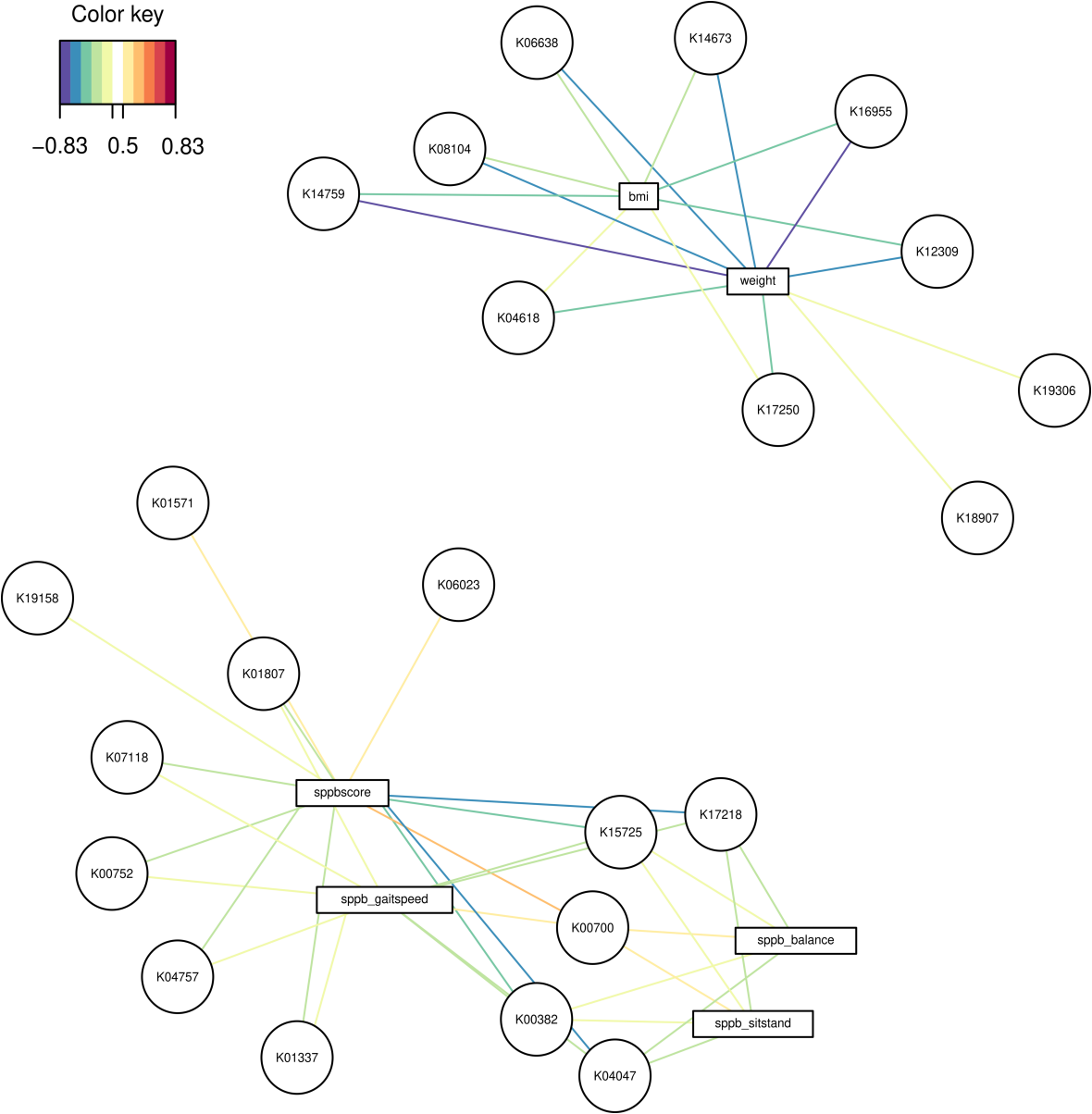

Supplement: Supplementary file 6 — Figure S6: Network map of the correlations between the KEGG functions and continuous clinical data. Spearman coefficient (ρ) equal to or greater than ±0.5 was used to plot the connections. [file ACEL-25-e70365-s001.pdf]

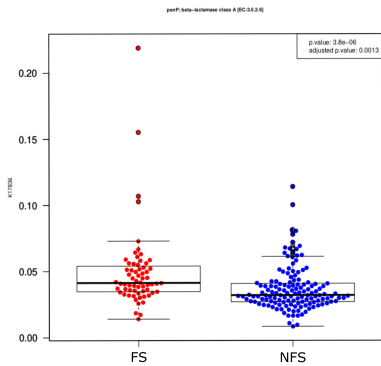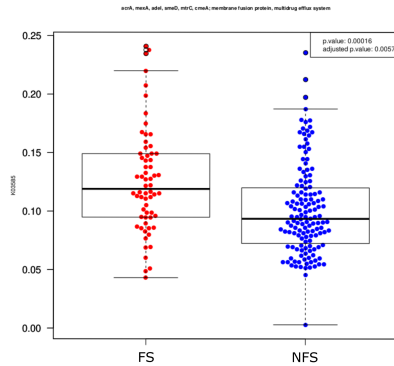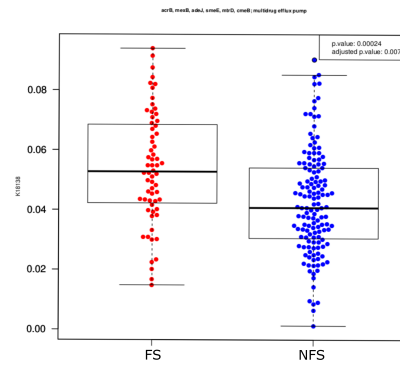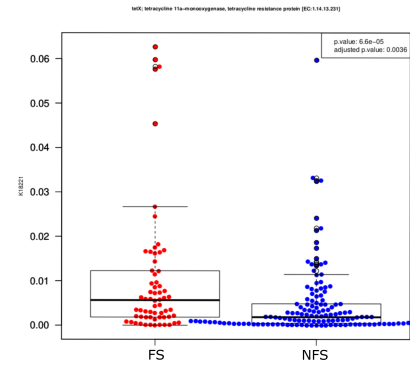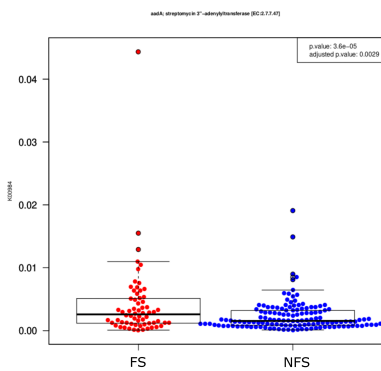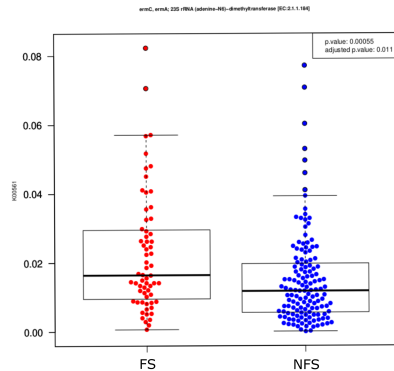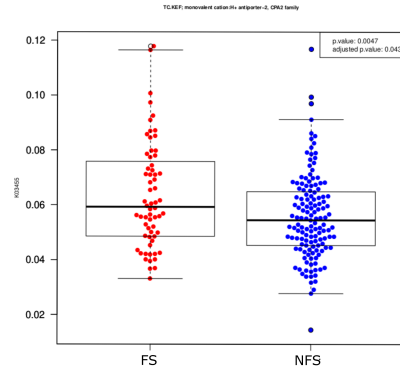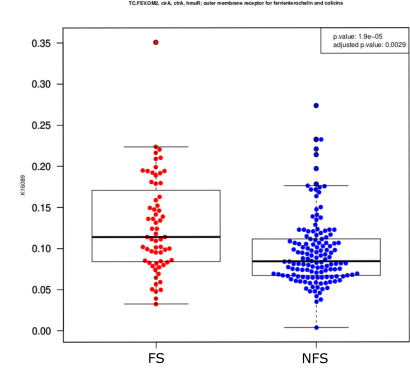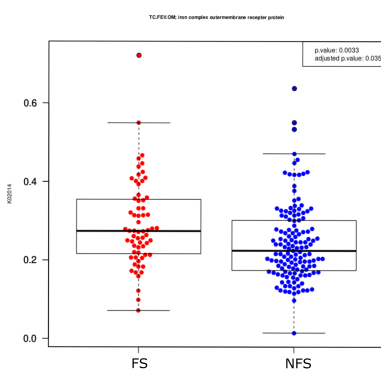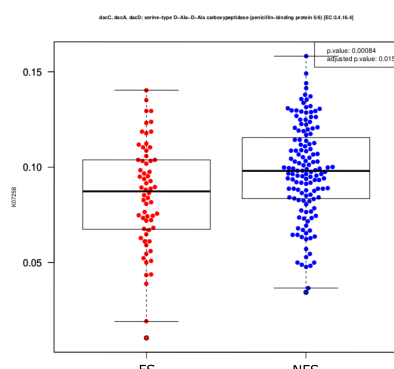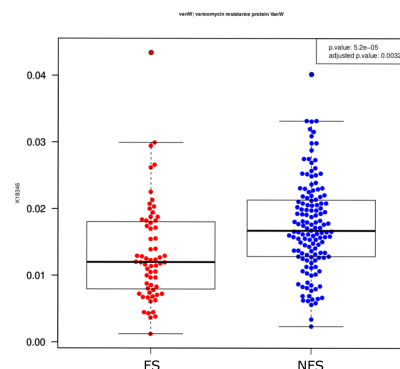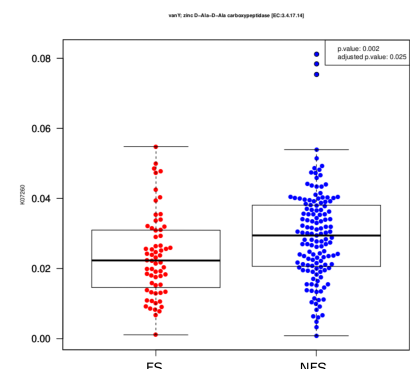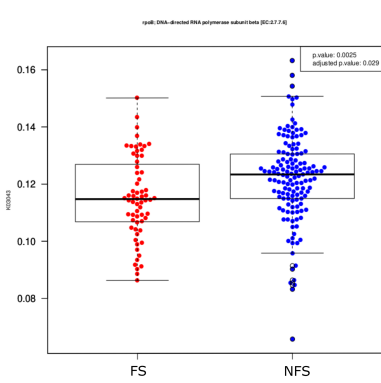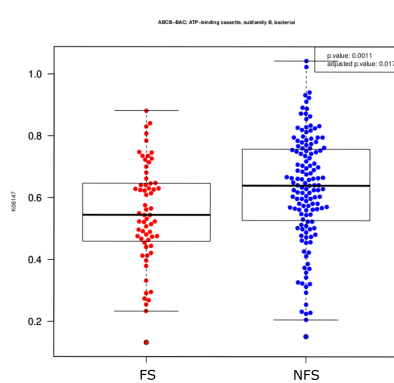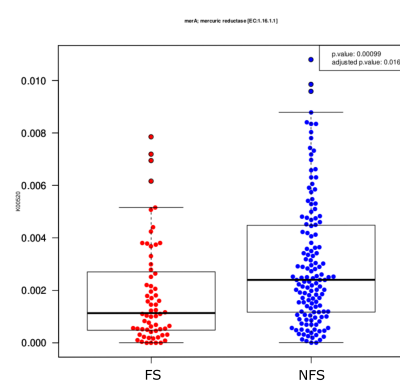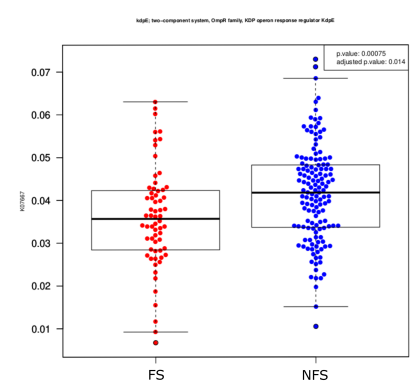

Supplement: Supplementary file 7 — Figure S7: Boxplots of the statistically significant KEGG functions involved in AR for the DESeq2 test between FS and NFS groups from both CARD and Reference Gene Catalog databases. FS, frail; NFS, non frail. [file ACEL-25-e70365-s008.pdf]
