# Supplementary material for: The Role of the Gut Microbiome in the Complex Network of Frailty Syndrome and Associated Comorbidities in Aging
Source: Aging Cell. 2026 Jan 10;25(2):e70365. doi: 10.1111/acel.70365 (PMC12790095; doi:10.1111/acel.70365)
Supplement: Supplementary file 2 — Figure S2: PCoA plots and Adonis test of the 16S taxonomic differences between groups for each categorical clinical feature. [file ACEL-25-e70365-s005.pdf]

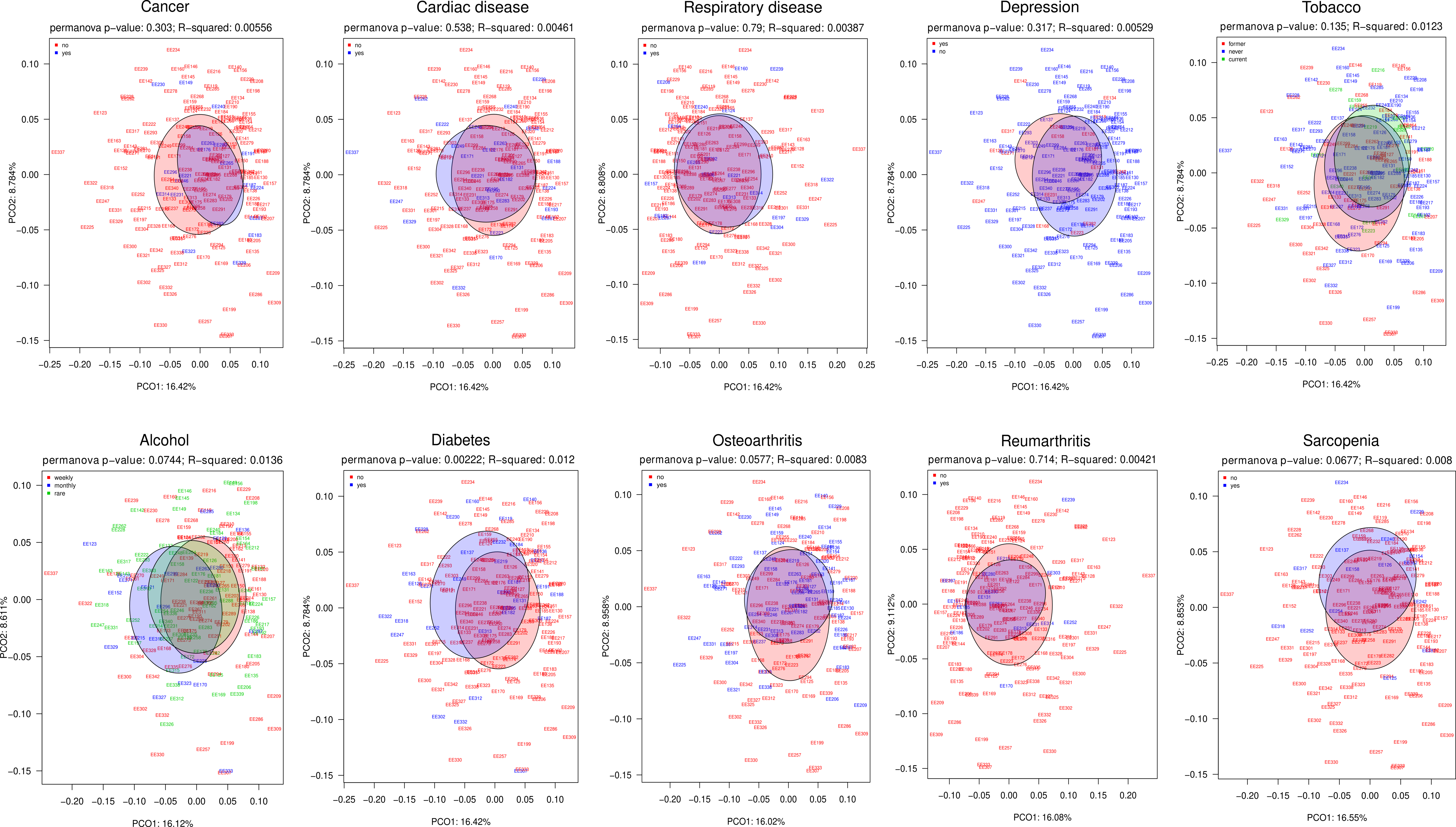
Cancer
permanova p−value: 0.303; R−squared: 0.00556
no
yes
PCO2: 8.784%
PCO1: 16.42%
Cardiac disease
permanova p−value: 0.538; R−squared: 0.00461
no
yes
PCO2: 8.784%
PCO1: 16.42%
Respiratory disease
permanova p−value: 0.79; R−squared: 0.00387
no
yes
PCO2: 8.808%
PCO1: 16.42%
Depression
permanova p−value: 0.317; R−squared: 0.00529
yes
no
PCO2: 8.784%
PCO1: 16.42%
Tobacco
permanova p−value: 0.135; R−squared: 0.0123
former
never
current
PCO2: 8.784%
PCO1: 16.42%
Alcohol
permanova p−value: 0.0744; R−squared: 0.0136
weekly
monthly
rare
PCO2: 8.611%
PCO1: 16.12%
Diabetes
permanova p−value: 0.00222; R−squared: 0.012
no
yes
PCO2: 8.784%
PCO1: 16.42%
Osteoarthritis
permanova p−value: 0.0577; R−squared: 0.0083
no
yes
PCO2: 8.958%
PCO1: 16.02%
Reumarthritis
permanova p−value: 0.714; R−squared: 0.00421
no
yes
PCO2: 9.112%
PCO1: 16.08%
Sarcopenia
permanova p−value: 0.0677; R−squared: 0.008
no
yes
PCO2: 8.853%
PCO1: 16.55%
