# Supplementary material for: The Role of the Gut Microbiome in the Complex Network of Frailty Syndrome and Associated Comorbidities in Aging
Source: Aging Cell. 2026 Jan 10;25(2):e70365. doi: 10.1111/acel.70365 (PMC12790095; doi:10.1111/acel.70365)
Supplement: Supplementary file 3 — Figure S3: PCoA plots and Adonis test of the functional differences between groups for each categorical clinical feature using KEGG functions data from shotgun sequencing. [file ACEL-25-e70365-s004.pdf]

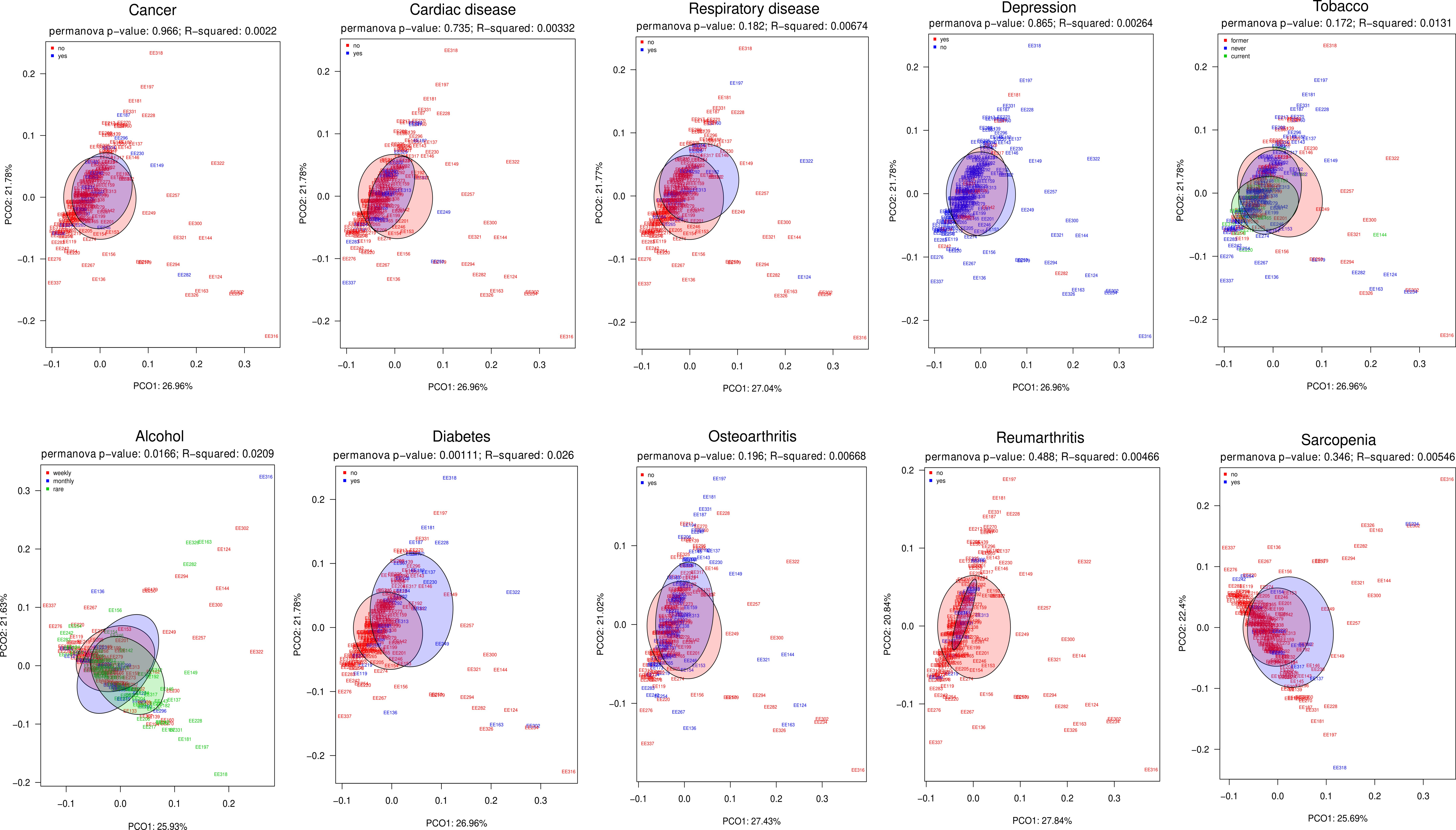
Cancer
permanova p−value: 0.966; R−squared: 0.0022
no
yes
PCO1: 26.96%
PCO2: 21.78%
Cardiac disease
permanova p−value: 0.735; R−squared: 0.00332
no
yes
PCO1: 26.96%
PCO2: 21.78%
Respiratory disease
permanova p−value: 0.182; R−squared: 0.00674
no
yes
PCO1: 27.04%
PCO2: 21.77%
Depression
permanova p−value: 0.865; R−squared: 0.00264
yes
no
PCO1: 26.96%
PCO2: 21.78%
Tobacco
permanova p−value: 0.172; R−squared: 0.0131
former
never
current
PCO1: 26.96%
PCO2: 21.78%
Alcohol
permanova p−value: 0.0166; R−squared: 0.0209
weekly
monthly
rare
PCO1: 25.93%
PCO2: 21.63%
Diabetes
permanova p−value: 0.00111; R−squared: 0.026
no
yes
PCO1: 26.96%
PCO2: 21.78%
Osteoarthritis
permanova p−value: 0.196; R−squared: 0.00668
no
yes
PCO1: 27.43%
PCO2: 21.02%
Reumarthritis
permanova p−value: 0.488; R−squared: 0.00466
no
yes
PCO1: 27.84%
PCO2: 20.84%
Sarcopenia
permanova p−value: 0.346; R−squared: 0.00546
no
yes
PCO1: 25.69%
PCO2: 22.4%
